# Supplementary material for: Contributions of mechanical loading and hormonal changes to eccentric hypertrophy during volume overload: A Bayesian analysis using logic-based network models
Source: PLoS Comput Biol. 2025 Apr 16;21(4):e1012390. doi: 10.1371/journal.pcbi.1012390 (PMC12040246; doi:10.1371/journal.pcbi.1012390)
Supplement: S1 Text — This document includes tables of the literature sources that provided data for each model input and output used in calibration and validation, more details on the fitting process used to generate probability density functions for inputs to the myocyte signaling network, and information on where covariance was present among the model inputs and how it was handled. (PDF) [file pcbi.1012390.s001.pdf]

Supplementary S1 Text

Contributions of mechanical loading and hormonal changes to eccentric hypertrophy during volume overload: a Bayesian analysis using a logic-based network cardiomyocyte model.

Johane H. Bracamonte[1], Lionel Watkins[2], Betty Pat [3,4], Louis J. Dell'Italia[3,4], Jeffrey J. Saucerman[2], Jeffrey W. Holmes[1,4,5,*]

(1) Department of Biomedical Engineering, University of Alabama at Birmingham, Birmingham, Alabama, United States of America.

(2) Department of Biomedical Engineering, University of Virginia, Charlottesville, Virginia, United States of America.

(3) Birmingham Veterans Affairs Health Care System, Birmingham, Alabama, United States of America.

(4) Division of Cardiovascular Disease, Heersink School of Medicine, University of Alabama at Birmingham, Birmingham, Alabama, United States of America.

(5) Division of Cardiothoracic Surgery, Heersink School of Medicine, University of Alabama at Birmingham, Birmingham, Alabama, United States of America.

* holmesjw@uab.edu

Sources of experimental data from canine MVR and experimental VO in rats used to fit the network model of cardiomyocyte hypertrophy.

Table A. Data sources for untreated VO.

Experimental measurement	References	
	Canine MVR	VO in rats
**Growth at cell and organ level**		
**LV mass/BW**	1–16	[17–33]
**LV EDV**	[2–8,11,14–16,34–45]	--
**Myocyte Cell Area/Length**	[3,5,10,11,16,39,42]	[17,46]
**Concentration in plasma of hypertrophy-related hormones and enzymes**		
**ANP**	[47–50]	[22,51–54]
**BNP**	[47–50]	[21,52]
**ANGII**	[11,12,47]	[21,24–26,51,55]
**NE**	[3,13,43,56]	[22,27,31,54,55,57]
**ET1**	[58,59]	[19,25]
**Intracellular signaling protein activity/phosphorylation**		
**FAK**	[1,7]	[60]
**Akt**	[1]	[61]
**ERK12**	[1]	[33,62]
**ERK5**	[44]	--
**JNK**	[1]	[62]
**p38**	[1,44]	[62]
**STAT**	--	[62]
**cGMP**	[34,44]	[51,63]
**ELK**	--	[33]
**Protein abundance**		
**ANP**		[23,52,64,65]
**BNP**	[40]	[23,52,64,65]
**αMHC**	[8,66]	[23,30,67]
**βMHC**		
**SERCA2**	[40]	[68,69]

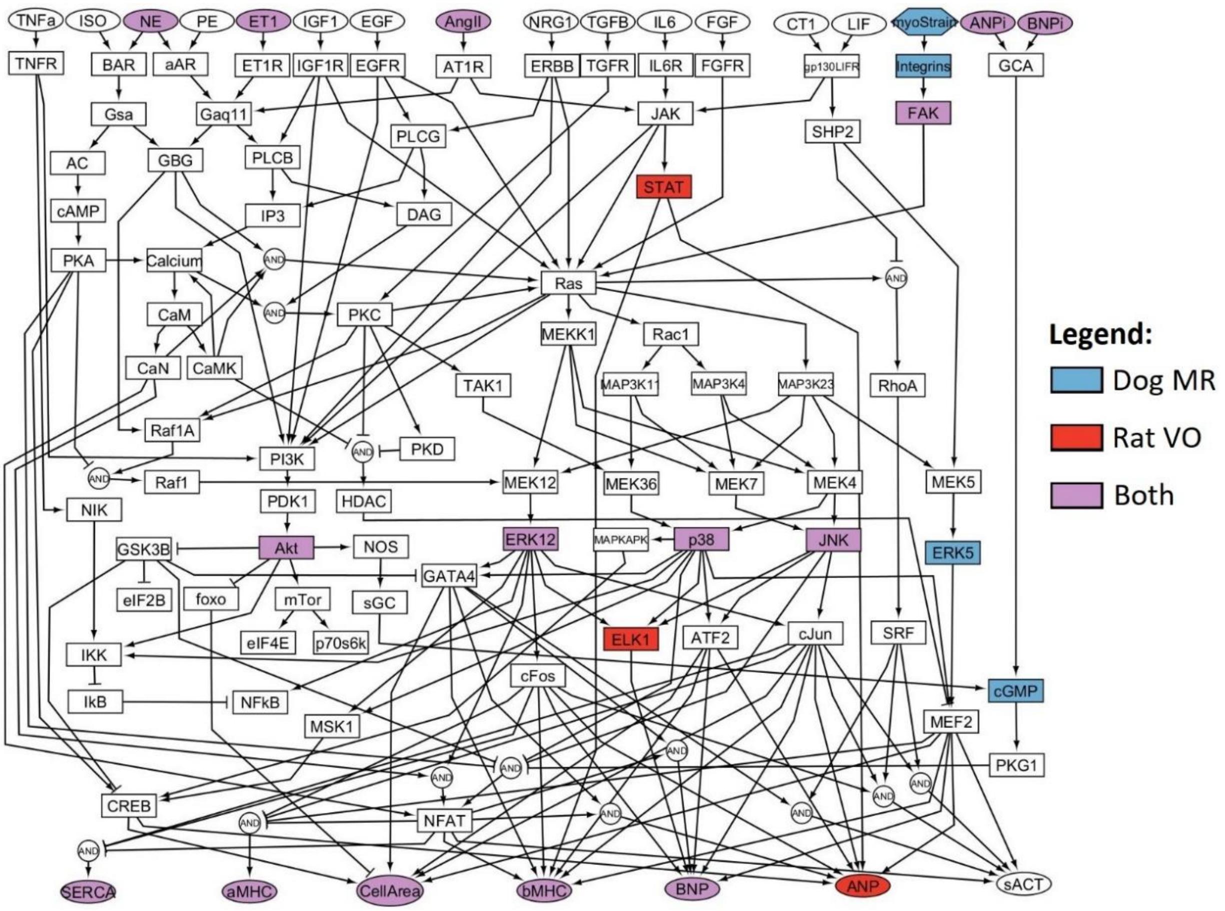

Fig A. Location in the network signaling model of available experimental data. See specific references in table A.

Table B. Data sources referenced in validation and discussion, not used for calibration.

Stimulus/Drug	Animal Model	Source
NE	Dog	[70–73]
ANGII	Rat	[74–87]
ISO	Rat	[88–99]
ISO+βB	Rat	[90,97,100,101]
ISO+ARB	Rat	[88,98,99,101,102]
NE+ERA	Rat	[103]
AngII+ERA	Rat	[87,104,105]
VO+βB	Dog	[1,16,34]
	Rat	[60,106]
VO+ARB	Dog	[11]
	Rat	[25,29,33]
VO+ERA	Rat	[107–110]
VO+ERA+ARB	Rat	[65,111]

Time-varying curves of neurohormonal alterations

We fitted the time course of variations in serum concentrations by allowing a step change immediately after the onset of VO and then fitting a continuous function over the remainder of

the time course. Visual inspection of integrated experimental data suggested that linear functions were sufficient to fit the time-varying fold changes of NE, ANP, and BNP beyond an initial step (if present). Beyond an initial step increase, fold changes in AngII concentration appeared consistent with an exponential decay. Finally, no discernable trend was observed in ET1 circulating concentration beyond the initial step, so we fitted for a single constant level for the remainder of the time course. We estimated the probability distribution of each function parameter with independent MCMC runs for each hormone, using the reported experimental concentrations to estimate the likelihood of each set of parameters.

Table C. PDFs for fitted parameters of functions describing neurohormonal alterations in VO.

Species	Function	a	b	C
ANP	$\frac{w_{ANP}}{w_{ANP}{}^{0}} = at/\tau + b$	1.0747 ± 0.5612	3.8311 ± 2.0542	--
BNP	$\frac{w_{BNP}}{w_{BNP}{}^{0}} = at/\tau + b$	1.5746 ± 0.2508	1.05 ± 0.05	--
ET1	$\frac{w_{ET1}}{w_{ET1}{}^{0}} = a$	3.0 ± 0.98	--	--
NE	$\frac{w_{NE}}{w_{NE}{}^{0}} = at/\tau + b$	0.1213 ± 0.0802	1.4610 ± 0.211	--
AngII	$\frac{w_{AngII}}{w_{AngII}{}^{0}} = ae^{-t/b} + c$	4.46 ± 0.98	0.533 ± 0.178	2.757 ± 0.793

Estimation of time-varying probability distributions of stretch

In a spherical model of the left ventricle, end-diastolic stretch can be calculated from the unloaded volume V_0 and the end-diastolic volume V_{ED} (see Section 2.4). If the LV is growing and remodeling, both of these volumes change over time. V_{ED} is typically measured and reported in overload experiments, while V_0 must be estimated from V_{ED} and other information. Designating individual time steps using the superscript *i*, this section outlines how we used available data to generate probability distributions for the time-varying values of end-diastolic and unloaded ventricular volumes $\left(V_{ED}^{i}, V_{0}^{i}\right)$ in dogs experiencing volume overload.

Step 1: We estimated the PDFs of unloaded ventricular dimensions at baseline, prior to the onset of volume overload. For this, we used reported data on end-diastolic volume, thickness, and maximum fiber stretch $(V_{ED}^{0}, h_{ED}^{0}, \lambda_{f}^{0})$ to calculate the unloaded volume and thickness at baseline (V_{0}^{0}, h_{0}^{0}) using the definition of stretch (Equation 2, main manuscript), the geometry of a thin-walled

sphere, and the assumption that the ventricular wall is incompressible, with LVM being written at any time step i (including i=0) as

$$LVM^i = \rho\left[\left(r_{ED}^i + h_{ED}^i\right)^3 - \left(r_{ED}^i\right)^3\right] = \rho\left[\left(r_0^i + h_0^i\right)^3 - \left(r_0^i\right)^3\right] \quad \text{(Equation S1.1)}$$

where ρ is the density of the myocardium that we assume to be constant. We used unrestricted random sampling of the inputs $(V_{ED}^0, h_{ED}^0, \lambda_f^0)$ to estimate the PDFs of the outputs (V_0^0, h_0^0) over 100,000 iterations using the conventional Monte Carlo method (Fig B).

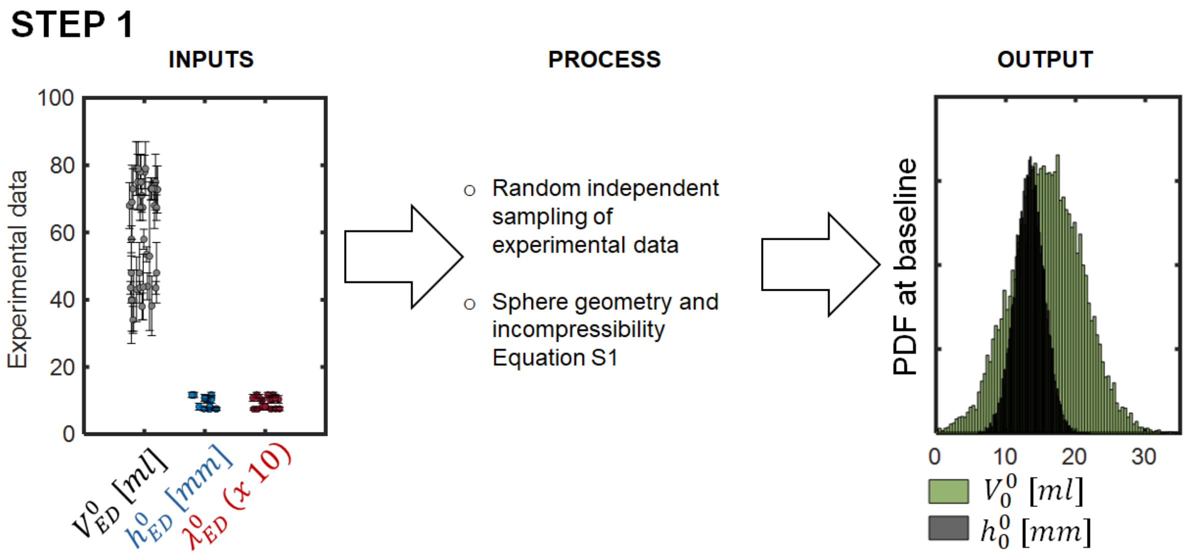

Fig B. Estimation of PDF of baseline unloaded ventricular dimensions from available experimental data from baseline end-diastolic dimensions.

Step 2: We estimated time-varying continuous PDFs of LVM and V_{ED} over the course of VO. For this, we assumed that changes in LV mass and end-diastolic diameter follow an exponential rise, consistent with the data shown in Figure 3:

$$\frac{LVM^i}{LVM^0} = C_M\left(1 - e^{\frac{t^i}{\tau_M}}\right) + 1 \quad \text{Equation S1.2}$$

$$\frac{V_{ED}^i}{V_{ED}^0} = \begin{cases} 1 & at\ \ t^i = 0 \\ C_V\left(1 - e^{\frac{t^i}{\tau_V}}\right) + D_V & at\ \ t^i > 0 \end{cases} \quad \text{Equation S1.3}$$

where t^i is the time at growth step i, and C_M, C_V, τ_M, τ_V, and D_V are empirical parameters fitted to $\frac{LVM^i}{LVM^0}$ and $\frac{V_{ED}^i}{V_{ED}^0}$ data derived from experimental measurements. Across multiple studies, larger animals with larger hearts at baseline will also have larger hearts following VO. We calculated the correlation between starting and ending masses and volumes during VO (PCC=0.87) using individual dog data reported by Ross et al. (1972) and Badke and Covell (1979) [112,113]. We

then used the method of Hayya et al. (1975) [114] for the division of two correlated and normally distributed variables to derive data on fold changes with respect to baseline $\left(\frac{LVM^i}{LVM^0}, \frac{V_{ED}^i}{V_{ED}^0}\right)$ from available experimental data $\left(LVM^0, V_{ED}^0, LVM^i, V_{ED}^i\right)$. We used random unrestricted sampling of exponential model parameters C_M, C_V, τ_M, τ_V, and D_V to create time-varying curves of fold changes of LVM and V_{ED}. On each iteration, these curves (and the associated parameters) were either retained or dropped based on their likelihood with respect to experimental data using the Metropolis-Hasting selection criteria in a classical MCMC setup. The result after 100,000 iterations is the PDFs of parameters C_M, C_V, τ_M, τ_V, and D_V that most likely reproduce the $\frac{LVM^i}{LVM^0}$ and $\frac{V_{ED}^i}{V_{ED}^0}$ data (Fig C).

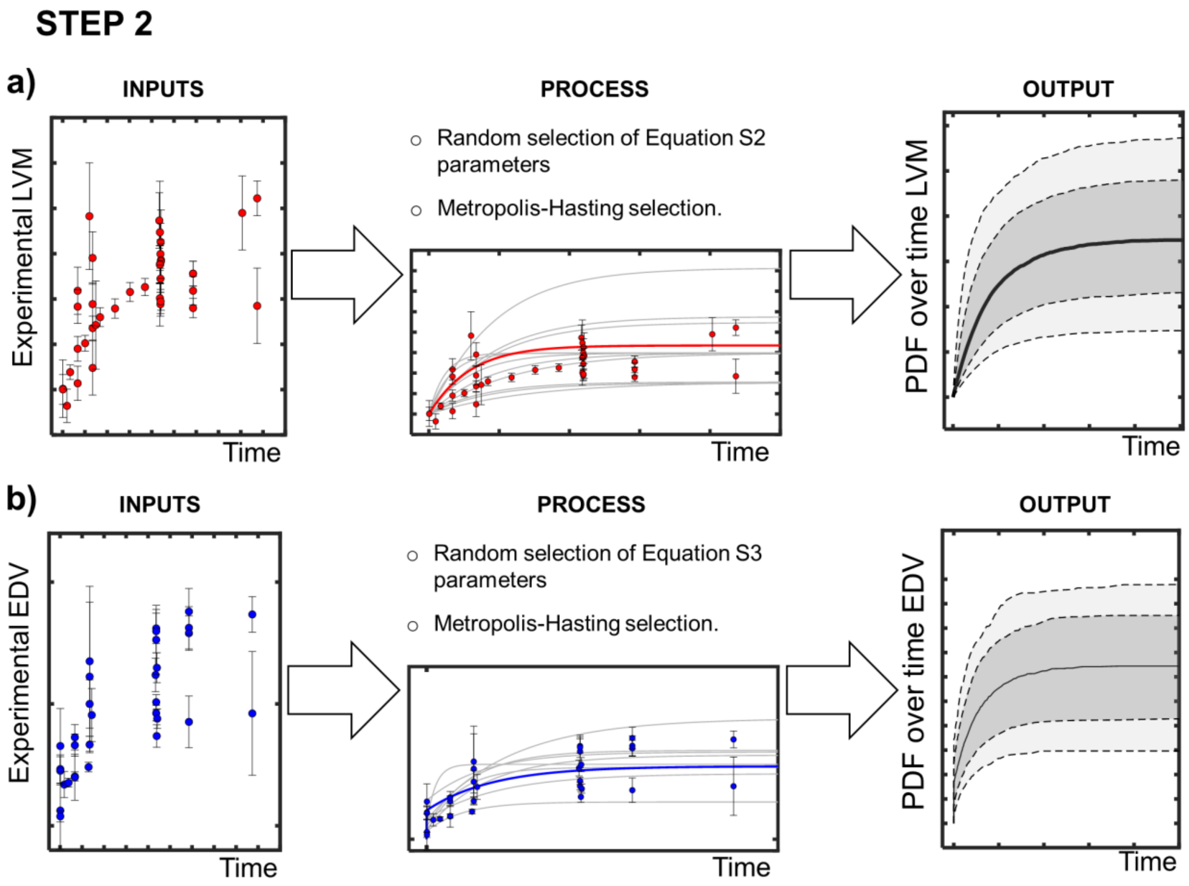

Fig C. Estimation of time-varying PDFs for parameters and associated exponential curves describing fold changes of LVM and EDV based on experimental data.

Step 3: Finally, we combined information from the previous steps to estimate the continuous time-varying PDF of stretch over the course of VO. First, we randomly selected a set of exponential parameters C_M, C_V, τ_M, τ_V, and D_V from their respective PDFs derived in Step 2. Those

parameters were either retained or dropped based on the joint likelihood of the associated chronic $\frac{LVM^i}{LVM^0}$ and $\frac{V_{ED}^i}{V_{ED}^0}$ with respect to experimental data using the Metropolis-Hasting selection criteria. The probability map of $\frac{LVM^i}{LVM^0}$ - $\frac{V_{ED}^i}{V_{ED}^0}$ combinations used to assess that joint likelihood was constructed from 24 experimental datasets from 16 studies reporting both quantities at in experimental MR in dogs [2–8,11,14,16,35,37,39,40,42,72]. As expected, experimentally measured increases in ventricular mass were directly correlated with simultaneously measured increases in end-diastolic volume (PCC=0.56), since both reflect the amount of eccentric hypertrophy that occurred. Following selection of a pair of mass and volume curves, we randomly selected baseline unloaded dimensions (V_0^0, h_0^0) from their PDFs derived in Step 1. We combined the baseline unloaded dimensions and the selected $\frac{LVM^i}{LVM^0}$ time curve to produce a time-varying curve of unloaded volume $\left(V_0^{\mathrm{i}}\right)$ over the course of VO by solving the following equation for r_0^{i},

$$\frac{LVM^i}{LVM^0} = \frac{\rho\left[\left(\, r_0^i + h_0^0\right)^3 - \left(r_0^i\right)^3\right]}{\rho[(r_0^0 + h_0^0)^3 - (\, r_0^0)^3]}, \qquad \text{Equation S1.4}$$

and calculating the unloaded volume $\left(V_0^i = \frac{4}{3}\pi r_0^i\right)$. Equation S1.4 arises directly from the geometry of a sphere (Equation S1.1) with the further assumption that during eccentric hypertrophy, all growth occurs in the radius / circumference of the sphere, with no change in wall thickness. We computed the time-varying curve for stretch for each iteration from the selected $\frac{V_{ED}^i}{V_{ED}^0}$ and computed V_0^{i} curves using Equation 2 (main manuscript) and generate the time-varying PDF for LV stretch by repeating this entire process over 100,000 iterations (Fig D).

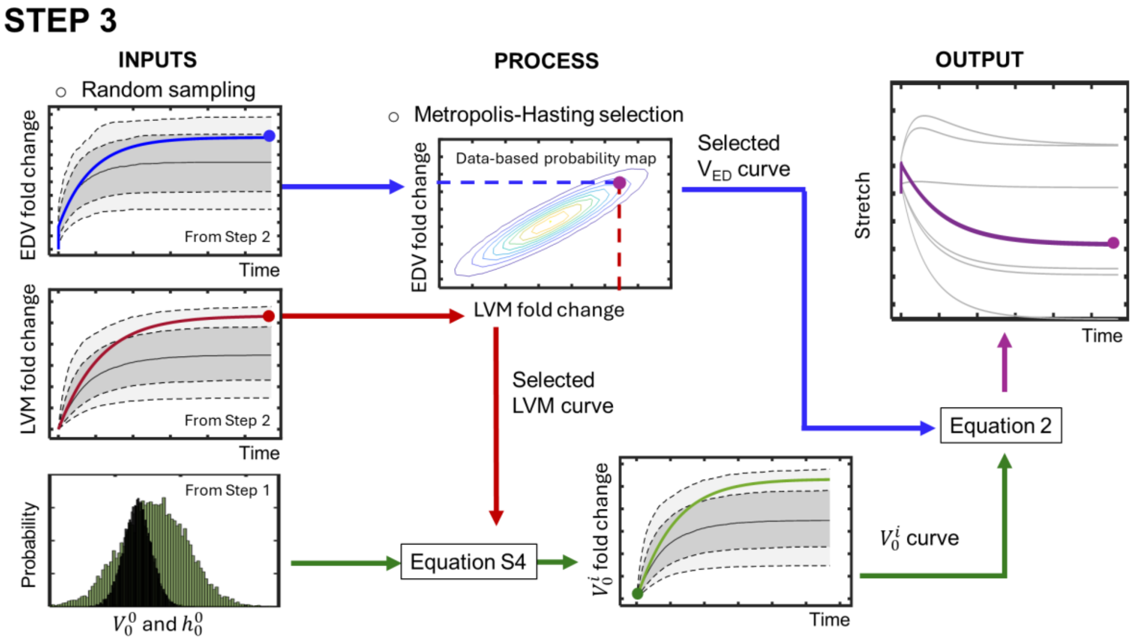

Fig D. Estimation of the time-varying probability distribution of ventricular stretch from PDFs of LVM, EDV and baseline unloaded dimensions.

The experimental correlations between baseline and chronic dimensions (employed in Step 2) [112,113] and between EDV and LVM growth (employed in Step 3) [2–8,11,14,16,35,37,39,40,42,72] are shown graphically in Fig E.

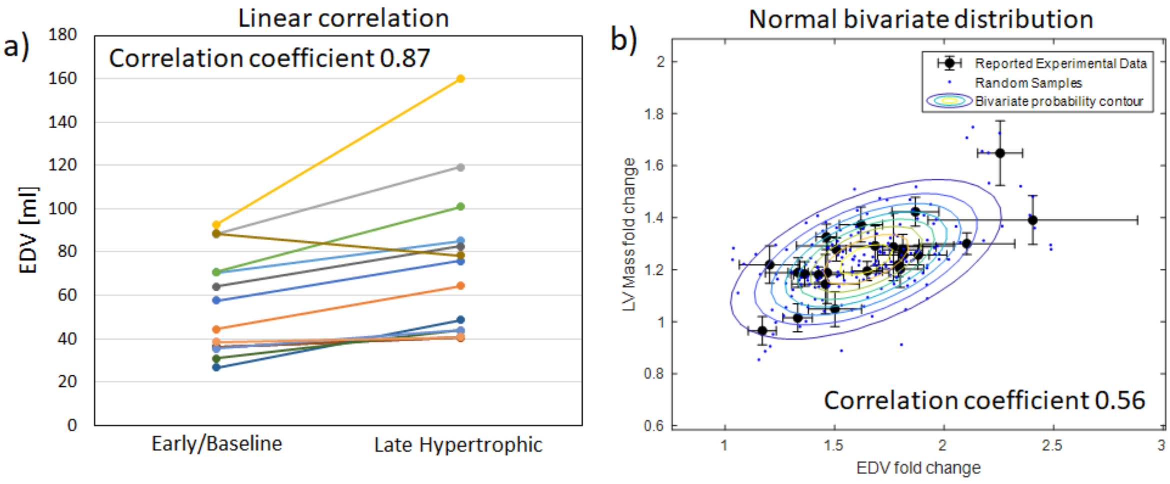

Fig E. Correlations considered in the Bayesian analysis of changes in LV stretch during VO. a) Correlation between baseline and chronic- volumes during experimental VO in dogs. b) Co-variance of growth as assessed by changes in ventricular mass and cavity volume from coupled data of experimental MR in dogs.

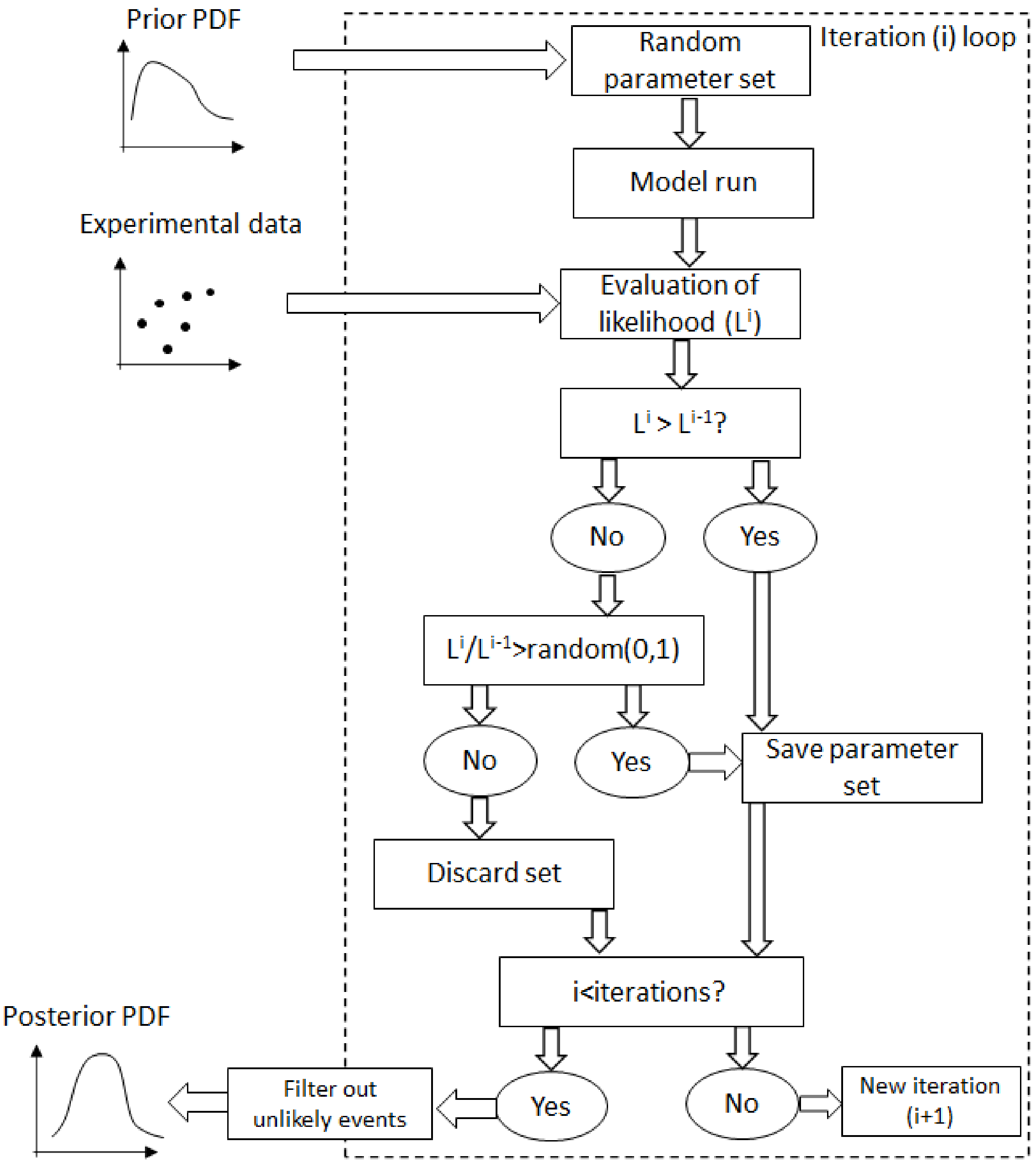

Fig F. Diagram of MCM algorithm with Metropolis-Hasting selection criteria.

Implementation of the Markov Chain Monte Carlo algorithm

The Markov Chain Monte Carlo (MCMC) algorithm with Metropolis Hasting selection criteria is a standard tool for Bayesian statistical analysis and machine learning. The algorithm utilizes known evidence of the behavior of a system to produce probability distributions for the model parameters (Fig F). The quality of the outputs of MCMC depends on the number of iterations of the chain. The number of iterations required to reach convergence depends on the specific problem. To

obtain reliable and reproduceable outputs we implemented the algorithm in two stages. In the first stage we assume a uniform probability distribution for all parameters and run 10,000 iterations. We use the outputs of the first stage as the prior probability distribution for the second stage and perform another 20,000 iterations with checks for convergence every 5,000 iterations. Convergence was reached for all runs before the end of the two-stage process (Fig G).

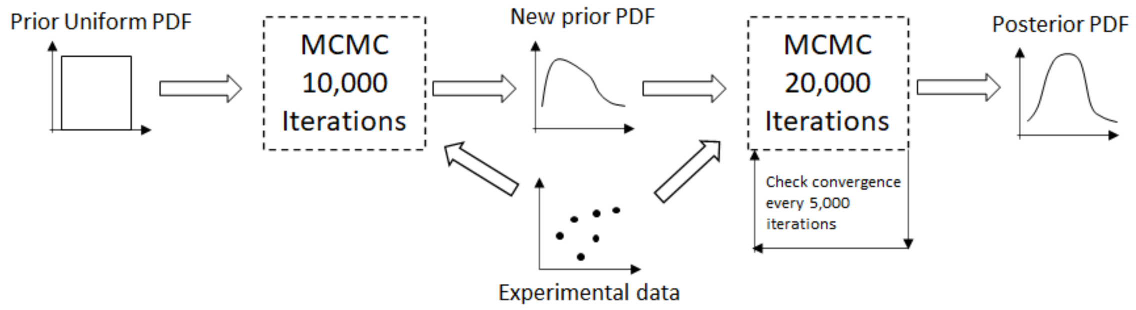

Fig G. MCMC implementation in two-stages for reproduceable convergence.

MCMC sampling and posterior parameter probability distributions of network parameters.

The CellArea node is sensitive to the weight of the myoStrain input, and this parameter heavily influenced the fraction of simulations that produced unlikely scenarios such as reverse growth and runaway growth. By repeating the MCMC analysis for several baseline myoStrain weights $(w^0_{myoStrain})$, we found that too many simulations showed runaway growth driven by ever-increasing NE levels for $w^0_{myoStrain} < 0.05$, while too many solutions showed unphysiological reversal of growth as strain declined at later time points for $w^0_{myoStrain} > 0.06$. We concluded that $0.05 < w^0_{myoStrain} < 0.06$ is the most likely range for this parameter (Fig H).

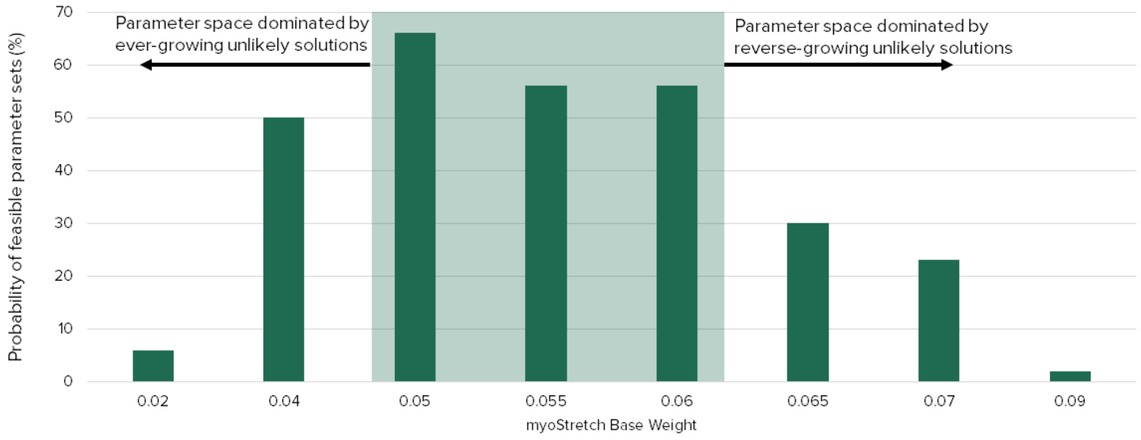

Fig H. Percentage of physiologically plausible solutions as a function of myoStrain input weights.

Within the subregion of most likely baseline input weights, each parameter was mostly independent of the others and displayed a nearly normal probability distribution. The strongest correlation (PCC=-0.42) identified was between the Background and ET1 baseline weights (Fig I).

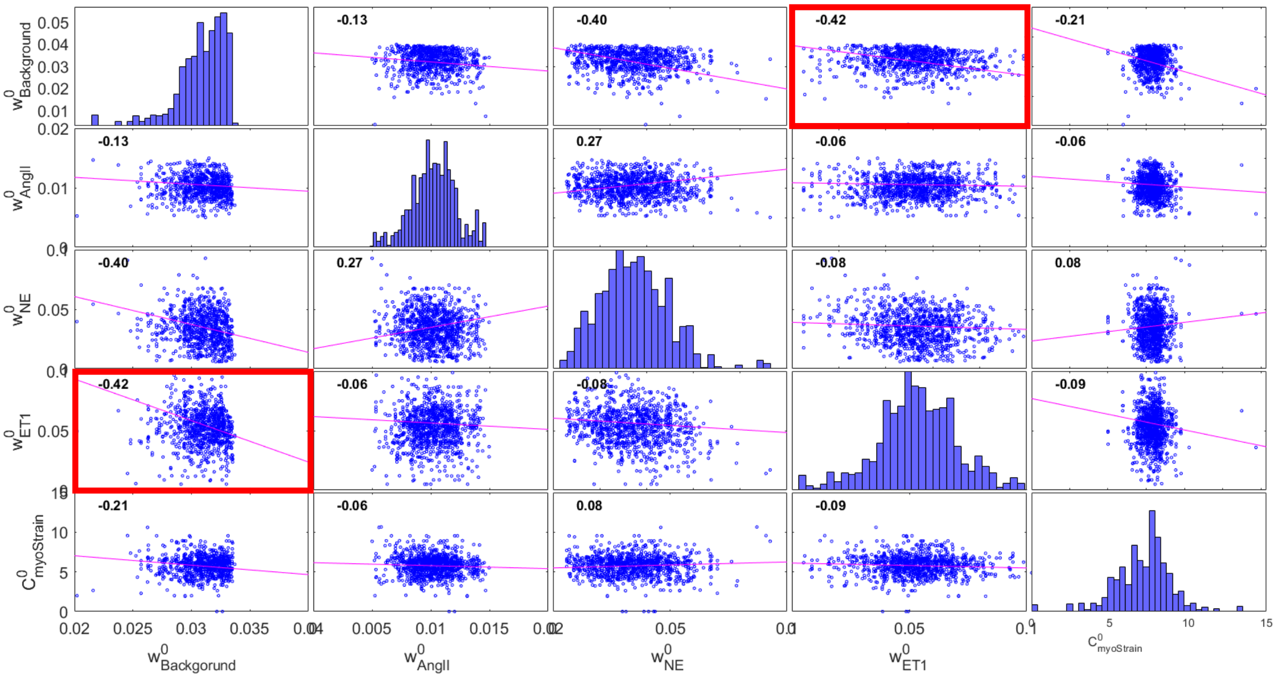

Fig I. Correlation matrix of baseline input weights and Strain mapping parameter after filtering-out unlikely results. The Pearson correlation coefficient PCC is shown for each pair of parameters to indicate correlation strength.

References

1. Sabri A, Rafiq K, Seqqat R, Kolpakov MA, Dillon R, Dell'italia LJ. Sympathetic Activation Causes Focal Adhesion Signaling Alteration in Early Compensated Volume Overload Attributable to Isolated Mitral Regurgitation in the Dog. *Circ Res*. 2008;102(9):1127-1136. doi:10.1161/CIRCRESAHA.107.163642

2. Nakano K, Swindle MM, Spinale F, et al. Depressed contractile function due to canine mitral regurgitation improves after correction of the volume overload. *J Clin Invest*. 1991;87(6):2077-2086. doi:10.1172/JCI115238

3. Tsutsui H, Spinale FG, Nagatsu M, et al. Effects of chronic beta-adrenergic blockade on the left ventricular and cardiocyte abnormalities of chronic canine mitral regurgitation. *Journal of Clinical Investigation*. 1994;93(6):2639-2648. doi:10.1172/JCI117277

4. Dell'Italia LJ, Meng QC, Balcells E, et al. Increased ACE and chymase-like activity in cardiac tissue of dogs with chronic mitral regurgitation. *American Journal of Physiology-Heart and Circulatory Physiology*. 1995;269(6):H2065-H2073. doi:10.1152/ajpheart.1995.269.6.H2065

5. Dell'italia LJ, Balcells E, Meng QC, et al. Volume-overload cardiac hypertrophy is unaffected by ACE inhibitor treatment in dogs. *American Journal of Physiology-Heart and Circulatory Physiology*. 1997;273(2):H961-H970. doi:10.1152/ajpheart.1997.273.2.H961

6. Katayama K, Tajimi T, Guth BD, et al. Early diastolic filling dynamics during experimental mitral regurgitation in the conscious dog. *Circulation*. 1988;78(2):390-400. doi:10.1161/01.CIR.78.2.390

7. Pat B, Chen Y, Killingsworth C, et al. Chymase inhibition prevents fibronectin and myofibrillar loss and improves cardiomyocyte function and LV torsion angle in dogs with isolated mitral regurgitation. *Circulation*. 2010;122(15):1488-1495. doi:10.1161/CIRCULATIONAHA.109.921619

8. Matsuo T, Carabello BA, Nagatomo Y, et al. Mechanisms of cardiac hypertrophy in canine volume overload. *American Journal of Physiology-Heart and Circulatory Physiology*. 1998;275(1):H65-H74. doi:10.1152/ajpheart.1998.275.1.H65

9. Carabello BA, Zile MR, Tanaka R, Cooper G. Left ventricular hypertrophy due to volume overload versus pressure overload. *American Journal of Physiology-Heart and Circulatory Physiology*. 1992;263(4):H1137-H1144. doi:10.1152/ajpheart.1992.263.4.H1137

10. Kihara Y, Sasayama S, Miyazaki S, et al. Role of the left atrium in adaptation of the heart to chronic mitral regurgitation in conscious dogs. *Circ Res*. 1988;62(3):543-553. doi:10.1161/01.RES.62.3.543

11. Perry GJ, Wei CC, Hankes GH, et al. Angiotensin II receptor blockade does not improve left ventricular function and remodeling in subacute mitral regurgitation in the dog. *J Am Coll Cardiol*. 2002;39(8):1374-1379. doi:10.1016/S0735-1097(02)01763-1

12. Tallaj J, Wei CC, Hankes GH, et al. β1-Adrenergic Receptor Blockade Attenuates Angiotensin II–Mediated Catecholamine Release Into the Cardiac Interstitium in Mitral

Regurgitation. *Circulation*. 2003;108(2):225-230. doi:10.1161/01.CIR.0000079226.48637.5A

13. Hankes GH, Ardell JL, Tallaj J, et al. β_1 <scp>-</scp> Adrenoceptor blockade mitigates excessive norepinephrine release into cardiac interstitium in mitral regurgitation in dog. *American Journal of Physiology-Heart and Circulatory Physiology*. 2006;291(1):H147-H151. doi:10.1152/ajpheart.00951.2005

14. Nemoto S, Razeghi P, Ishiyama M, De Freitas G, Taegtmeyer H, Carabello BA. PPAR-γ agonist rosiglitazone ameliorates ventricular dysfunction in experimental chronic mitral regurgitation. *American Journal of Physiology-Heart and Circulatory Physiology*. 2005;288(1):H77-H82. doi:10.1152/ajpheart.01246.2003

15. Nemoto S, Hamawaki M, De Freitas G, Carabello BA. differential effects of the angiotensin-converting enzyme inhibitor lisinopril versus the beta-adrenergic receptor blocker atenolol on hemodynamics and left ventricular contractile function in experimental mitral regurgitation. *J Am Coll Cardiol*. 2002;40(1):149-154. doi:10.1016/S0735-1097(02)01926-5

16. Pat B, Killingsworth C, Denney T, et al. Dissociation between cardiomyocyte function and remodeling with β-adrenergic receptor blockade in isolated canine mitral regurgitation. *American Journal of Physiology-Heart and Circulatory Physiology*. 2008;295(6):H2321-H2327. doi:10.1152/ajpheart.00746.2008

17. Jobe LJ, Meléndez GC, Levick SP, Du Y, Brower GL, Janicki JS. TNF-α inhibition attenuates adverse myocardial remodeling in a rat model of volume overload. *American Journal of Physiology-Heart and Circulatory Physiology*. 2009;297(4):H1462-H1468. doi:10.1152/ajpheart.00442.2009

18. Lu H, Meléndez GC, Levick SP, Janicki JS. Prevention of adverse cardiac remodeling to volume overload in female rats is the result of an estrogen-altered mast cell phenotype. *American Journal of Physiology-Heart and Circulatory Physiology*. 2012;302(3):H811-H817. doi:10.1152/ajpheart.00980.2011

19. Gardner JD, Murray DB, Voloshenyuk TG, Brower GL, Bradley JM, Janicki JS. Estrogen attenuates chronic volume overload induced structural and functional remodeling in male rat hearts. *American Journal of Physiology-Heart and Circulatory Physiology*. 2010;298(2):H497-H504. doi:10.1152/ajpheart.00336.2009

20. Mclarty JL, Meléndez GC, Levick SP, et al. Estrogenic modulation of inflammation-related genes in male rats following volume overload. *Physiol Genomics*. 2012;44(6):362-373. doi:10.1152/PHYSIOLGENOMICS.00146.2011/SUPPL_FILE/SUPPMAT.PDF

21. Wang XH, Zhuo XZ, Ni YJ, et al. Improvement of cardiac function and reversal of gap junction remodeling by Neuregulin-1β in volume-overloaded rats with heart failure. *J Geriatr Cardiol*. 2012;9(2):172. doi:10.3724/SP.J.1263.2012.03271

22. Willenbrock R, Stauss H, Scheuermann M, Osterziel KJ, Unger T, Dietz R. Effect of chronic volume overload on baroreflex control of heart rate and sympathetic nerve activity. *Am J Physiol*. 1997;273(6):H2580-5. doi:10.1152/ajpheart.1997.273.6.H2580

23. Lachance D, Dhahri W, Drolet MC, et al. Endurance training or beta-blockade can partially block the energy metabolism remodeling taking place in experimental chronic left ventricle volume overload. *BMC Cardiovasc Disord*. 2014;14(1):190. doi:10.1186/1471-2261-14-190

24. Shuji H, Takuroh I, Takeshi M, et al. Differential responses of circulating and tissue adrenomedullin and gene expression to volume overload. *J Card Fail*. 2000;6(2):120-129. doi:10.1016/S1071-9164(00)90014-9

25. Ishiye M, Umemura K, Uematsu T, Nakashima M. Effects of Losartan, an Angiotensin II Antagonist, on the Development of Cardiac Hypertrophy Due to Volume Overload. *Biol Pharm Bull*. 1995;18(5):700-704. doi:10.1248/BPB.18.700

26. Bauer P, Regitz-Zagrosek V, Kallisch H, et al. Myocardial angiotensin receptor type 1 gene expression in a rat model of cardiac volume overload. *Basic Res Cardiol*. 1997;92(3):139-146. doi:10.1007/BF00788631/METRICS

27. Oka T, Nishimura H, Ueyama M, Kubota J, Kawamura K. Lisinopril reduces cardiac hypertrophy and mortality in rats with aortocaval fistula. *Eur J Pharmacol*. 1993;234(1):55-60. doi:10.1016/0014-2999(93)90705-M

28. Isgaard J, Wåhlander H, Adams MA, Friberg P. Increased expression of growth hormone receptor mRNA and insulin-like growth factor-I mRNA in volume-overloaded hearts. *Hypertension*. 1994;23(6):884-888. doi:10.1161/01.HYP.23.6.884

29. Ruzicka M, Yuan B, Harmsen E, Leenen FH. The renin-angiotensin system and volume overload-induced cardiac hypertrophy in rats. Effects of angiotensin converting enzyme inhibitor versus angiotensin II receptor blocker. *Circulation*. 1993;87(3):921-930. doi:10.1161/01.CIR.87.3.921

30. Pu M, Gao Z, Zhang X, et al. Impact of mitral regurgitation on left ventricular anatomic and molecular remodeling and systolic function: implication for outcome. *American Journal of Physiology-Heart and Circulatory Physiology*. 2009;296(6):H1727-H1732. doi:10.1152/ajpheart.00882.2008

31. Kristen A V., Kreusser MM, Lehmann L, et al. Preserved Norepinephrine Reuptake but Reduced Sympathetic Nerve Endings in Hypertrophic Volume-Overloaded Rat Hearts. *J Card Fail*. 2006;12(7):577-583. doi:10.1016/J.CARDFAIL.2006.05.006

32. Stefano LM De, Matsubara LS, Matsubara BB. Myocardial dysfunction with increased ventricular compliance in volume overload hypertrophy. *Eur J Heart Fail*. 2006;8(8):784-789. doi:10.1016/J.EJHEART.2006.02.005

33. Zhang W, Elimban V, Xu YJ, Zhang M, Nijjar MS, Dhalla NS. Alterations of Cardiac ERK1/2 Expression and Activity Due to Volume Overload Were Attenuated by the Blockade of RAS. *J Cardiovasc Pharmacol Ther*. 2010;15(1):84-92. doi:10.1177/1074248409356430

34. Trappanese DM, Liu Y, McCormick RC, et al. Chronic β1-adrenergic blockade enhances myocardial β3-adrenergic coupling with nitric oxide-cGMP signaling in a canine model of chronic volume overload: new insight into mechanisms of cardiac benefit with selective β1-blocker therapy. *Basic Res Cardiol*. 2015;110(1):456. doi:10.1007/s00395-014-0456-3

35. Kleaveland JP, Kussmaul WG, Vinciguerra T, Diters R, Carabello BA. Volume overload hypertrophy in a closed-chest model of mitral regurgitation. *American Journal of Physiology-Heart and Circulatory Physiology*. 1988;254(6):H1034-H1041. doi:10.1152/ajpheart.1988.254.6.H1034

36. Nakano K, Sugawara M, Ishihara K, et al. Myocardial stiffness derived from end-systolic wall stress and logarithm of reciprocal of wall thickness. Contractility index independent of ventricular size. *Circulation*. 1990;82(4):1352-1361. doi:10.1161/01.CIR.82.4.1352

37. Carabello BA, Nakano K, Corin W, Biederman R, Spann JF. Left ventricular function in experimental volume overload hypertrophy. *American Journal of Physiology-Heart and Circulatory Physiology*. 1989;256(4):H974-H981. doi:10.1152/ajpheart.1989.256.4.H974

38. Berko B, Gaasch WH, Tanigawa N, Smith D, Craige E. Disparity between ejection and end-systolic indexes of left ventricular contractility in mitral regurgitation. *Circulation*. 1987;75(6):1310-1319. doi:10.1161/01.CIR.75.6.1310

39. Spinale FG, Ishihra K, Zile M, DeFryte G, Crawford FA, Carabello BA. Structural basis for changes in left ventricular function and geometry because of chronic mitral regurgitation and after correction of volume overload. *J Thorac Cardiovasc Surg*. 1993;106(6):1147-1157. doi:10.1016/S0022-5223(19)33992-3

40. Zheng J, Chen Y, Pat B, et al. Molecular cardiology microarray identifies extensive downregulation of noncollagen extracellular matrix and profibrotic growth factor genes in chronic isolated mitral regurgitation in the dog. *Circulation*. 2009;119(15):2086-2095. doi:10.1161/CIRCULATIONAHA.108.826230

41. Carabello BA, Nakano K, Ishihara K, Kanazawa S, W Biederman RW, Spann Jr JF. Coronary blood flow in dogs with contractile dysfunction due to experimental volume overload. *Circulation*. 1991;83(3):1063-1075. doi:10.1161/01.CIR.83.3.1063

42. Urabe Y, Mann DL, Kent RL, et al. Cellular and ventricular contractile dysfunction in experimental canine mitral regurgitation. *Circ Res*. 1992;70(1):131-147. doi:10.1161/01.RES.70.1.131

43. Lee JD, Sasayama S, Kihara Y, et al. Adaptations of the left ventricle to chronic volume overload induced by mitral regurgitation in conscious dogs. *Heart Vessels*. 1985;1(1):9-15. doi:10.1007/BF02066481/METRICS

44. Liu Y, Dillon AR, Tillson M, et al. Volume overload induces differential spatiotemporal regulation of myocardial soluble guanylyl cyclase in eccentric hypertrophy and heart failure. *J Mol Cell Cardiol*. 2013;60(1):72-83. doi:10.1016/J.YJMCC.2013.03.019

45. Stewart JA, Wei CC, Brower GL, et al. Cardiac mast cell- and chymase-mediated matrix metalloproteinase activity and left ventricular remodeling in mitral regurgitation in the dog. *J Mol Cell Cardiol*. 2003;35(3):311-319. doi:10.1016/S0022-2828(03)00013-0

46. Du Y, Plante E, Janicki JS, Brower GL. Temporal Evaluation of Cardiac Myocyte Hypertrophy and Hyperplasia in Male Rats Secondary to Chronic Volume Overload. *Am J Pathol*. 2010;177(3):1155-1163. doi:10.2353/ajpath.2010.090587

47. Ichiki T, Boerrigter G, Huntley BK, et al. Differential expression of the pro-natriuretic peptide convertases corin and furin in experimental heart failure and atrial fibrosis. *American Journal of Physiology-Regulatory, Integrative and Comparative Physiology*. 2013;304(2):R102-R109. doi:10.1152/ajpregu.00233.2012

48. Asano K, Masuda K, Okumura M, Kadosawa T, Fujinaga T. Plasma Atrial and Brain Natriuretic Peptide Levels in Dogs with Congestive Heart Failure. *Journal of Veterinary Medical Science*. 1999;61(5):523-529. doi:10.1292/jvms.61.523

49. Asano K, Masuda K, Okumura M, Kadosawa T, Fujinaga T. Association between Exogenous Atrial Natriuretic Peptide and Hemodynamics in Dogs with Congestive Heart Failure Produced by Experimental Mitral Regurgitation. *Journal of Veterinary Medical Science*. 2001;63(3):243-250. doi:10.1292/JVMS.63.243

50. Hori Y, Sano N, Kanai K, Hoshi F, Itoh N, Higuchi SI. Acute cardiac volume load-related changes in plasma atrial natriuretic peptide and N-terminal pro-B-type natriuretic peptide concentrations in healthy dogs. *The Veterinary Journal*. 2010;185(3):317-321. doi:10.1016/J.TVJL.2009.06.008

51. Pagel I, Langenickel T, Höhnel K, et al. Cardiac and renal effects of growth hormone in volume overload-induced heart failure: role of NO. *Hypertension*. 2002;39(1):57-62. doi:10.1161/HY0102.098323

52. Langenickel T, Pagel I, Höhnel K, Dietz R, Willenbrock R. Differential regulation of cardiac ANP and BNP mRNA in different stages of experimental heart failure. *American Journal of Physiology-Heart and Circulatory Physiology*. 2000;278(5):H1500-H1506. doi:10.1152/ajpheart.2000.278.5.H1500

53. Abassi Z, Goltsman I, Karram T, Winaver J, Hoffman A. Aortocaval Fistula in Rat: A Unique Model of Volume-Overload Congestive Heart Failure and Cardiac Hypertrophy. Gualillo O, ed. *Biomed Res Int*. 2011;2011(1). doi:10.1155/2011/729497

54. Huang M, Hester RL, Guyton AC. Hemodynamic changes in rats after opening an arteriovenous fistula. *https://doi.org/101152/ajpheart19922623H846*. 1992;262(3 31-3). doi:10.1152/AJPHEART.1992.262.3.H846

55. Suzuki H, Maehara K, Yaoita H, Maruyama Y. Altered Effects of Angiotensin II Type 1 and Type 2 Receptor Blockers on Cardiac Norepinephrine Release and Inotropic Responses During Cardiac Sympathetic Nerve Stimulation in Aorto-Caval Shunt Rats. *Circulation Journal*. 2004;68(7):683-690. doi:10.1253/CIRCJ.68.683

56. Nagatsu M, Zile MR, Tsutsui H, et al. Native beta-adrenergic support for left ventricular dysfunction in experimental mitral regurgitation normalizes indexes of pump and contractile function. *Circulation*. 1994;89(2):818-826. doi:10.1161/01.CIR.89.2.818

57. Kristen A V., Just A, Haass M, Seller H. Central hypercapnic chemoreflex modulation of renal sympathetic nerve activity in experimental heart failure. *Basic Res Cardiol*. 2002;97(2):177-186. doi:10.1007/S003950200009/METRICS

58. Ray L, Mathieu M, Jespers P, et al. Early increase in pulmonary vascular reactivity with overexpression of endothelin-1 and vascular endothelial growth factor in canine

experimental heart failure. *Exp Physiol*. 2008;93(3):434-442. doi:10.1113/EXPPHYSIOL.2007.040469

59. Cavero PG, Miller WL, Heublein DM, Margulies KB, Burnett JC. Endothelin in experimental congestive heart failure in the anesthetized dog. *American Journal of Physiology-Renal Physiology*. 1990;259(2):F312-F317. doi:10.1152/ajprenal.1990.259.2.F312

60. Seqqat R, Guo X, Rafiq K, et al. Beta1-adrenergic receptors promote focal adhesion signaling downregulation and myocyte apoptosis in acute volume overload. *J Mol Cell Cardiol*. 2012;53(2):240-249. doi:10.1016/j.yjmcc.2012.05.004

61. Dent MR, Das S, Dhalla NS. Alterations in both death and survival signals for apoptosis in heart failure due to volume overload. *J Mol Cell Cardiol*. 2007;43(6):726-732. doi:10.1016/J.YJMCC.2007.09.001

62. Kolpakov MA, Seqqat R, Rafiq K, et al. Pleiotropic effects of neutrophils on myocyte apoptosis and left ventricular remodeling during early volume overload. *J Mol Cell Cardiol*. 2009;47(5):634-645. doi:10.1016/J.YJMCC.2009.08.016

63. Arnal JF, Philippe M, Laboulandine I, Michel JB. Effect of perindopril in rat cardiac volume overload. *Am Heart J*. 1993;126(3):776-782. doi:10.1016/0002-8703(93)90929-4

64. Yamakawa H, Imamura T, Matsuo T, et al. Diastolic wall stress and ANG II in cardiac hypertrophy and gene expression induced by volume overload. *American Journal of Physiology-Heart and Circulatory Physiology*. 2000;279(6):H2939-H2946. doi:10.1152/ajpheart.2000.279.6.H2939

65. Fareh J, Touyz RM, Schiffrin EL, Thibault G. Endothelin-1 and Angiotensin II Receptors in Cells From Rat Hypertrophied Heart. *Circ Res*. 1996;78(2):302-311. doi:10.1161/01.RES.78.2.302

66. Imamura T, McDermott PJ, Kent RL, Nagatsu M, Cooper IV G, Carabello BA. Acute changes in myosin heavy chain synthesis rate in pressure versus volume overload. *Circ Res*. 1994;75(3):418-425. doi:10.1161/01.RES.75.3.418

67. Freire G, Ocampo C, Ilbawi N, Griffin AJ, Gupta M. Overt expression of AP-1 reduces alpha myosin heavy chain expression and contributes to heart failure from chronic volume overload. *J Mol Cell Cardiol*. 2007;43(4):465-478. doi:10.1016/J.YJMCC.2007.07.046

68. Pu M, Gao Z, Pu DK, Davidson WR. Effects of early, late, and long-term nonselective β-blockade on left ventricular remodeling, function, and survival in chronic organic mitral regurgitation. *Circ Heart Fail*. 2013;6(4):756-762. doi:10.1161/CIRCHEARTFAILURE.112.000196

69. Wojciechowski P, Juric D, Louis XL, et al. Resveratrol Arrests and Regresses the Development of Pressure Overload- but Not Volume Overload-Induced Cardiac Hypertrophy in Rats. *J Nutr*. 2010;140(5):962-968. doi:10.3945/JN.109.115006

70. King BD, Sack D, Kichuk MR, Hintze TH. Absence of hypertension despite chronic marked elevations in plasma norepinephrine in conscious dogs. *Hypertension*. 1987;9(6):582-590. doi:10.1161/01.HYP.9.6.582

71. Laks MM, Morady F, Swan HJC. Myocardial Hypertrophy Produced by Chronic Infusion of Subhypertensive Doses of Norepinephrine in the Dog. *Chest*. 1973;64(1):75-78. doi:10.1378/chest.64.1.75

72. Stewart JM, Patel MB, Wang J, et al. Chronic elevation of norepinephrine in conscious dogs produces hypertrophy with no loss of LV reserve. *American Journal of Physiology-Heart and Circulatory Physiology*. 1992;262(2):H331-H339. doi:10.1152/ajpheart.1992.262.2.H331

73. Raum WJ, Laks MM, Garner D, Swerdloff RS. β-Adrenergic receptor and cyclic AMP alterations in the canine ventricular septum during long-term norepinephrine infusion: Implications for hypertrophic cardiomyopathy. *Circulation*. 1983;68(3 I):693-699. doi:10.1161/01.CIR.68.3.693

74. Griffin SA, Brown WCB, MacPherson F, et al. Angiotensin II causes vascular hypertrophy in part by a non-pressor mechanism. *Hypertension*. 1991;17(5):626-635. doi:10.1161/01.HYP.17.5.626

75. Dostal DE, Baker KM. Angiotensin II Stimulation of Left Ventricular Hypertrophy in Adult Rat HeartMediation by the AT1 Receptor. *Am J Hypertens*. 1992;5(5_Pt_1):276-280. doi:10.1093/AJH/5.5.276

76. Dilley R. Heparin inhibits mesenteric vascular hypertrophy in angiotensin II-infusion hypertension in rats. *Cardiovasc Res*. 1998;38(1):247-255. doi:10.1016/S0008-6363(98)00004-2

77. Baltatu O, Silva JA, Ganten D, Bader M. The Brain Renin-Angiotensin System Modulates Angiotensin II–Induced Hypertension and Cardiac Hypertrophy. *Hypertension*. 2000;35(1):409-412. doi:10.1161/01.HYP.35.1.409

78. Kim S, Ohta K, Hamaguchi A, Yukimura T, Miura K, Iwao H. Angiotensin II Induces Cardiac Phenotypic Modulation and Remodeling In Vivo in Rats. *Hypertension*. 1995;25(6):1252-1259. doi:10.1161/01.HYP.25.6.1252

79. Mishra JS, More AS, Gopalakrishnan K, Kumar S. Testosterone plays a permissive role in angiotensin II-induced hypertension and cardiac hypertrophy in male rats. *Biol Reprod*. 2019;100(1):139-148. doi:10.1093/BIOLRE/IOY179

80. Grobe JL, Mecca AP, Lingis M, et al. Prevention of angiotensin II-induced cardiac remodeling by angiotensin-(1–7). *American Journal of Physiology-Heart and Circulatory Physiology*. 2007;292(2):H736-H742. doi:10.1152/ajpheart.00937.2006

81. Goldspink PH, McKinney RD, Kimball VA, Geenen DL, Buttrick PM. Angiotensin II induced cardiac hypertrophy in vivo is inhibited by cyclosporin A in adult rats. *Mol Cell Biochem*. 2001;226(1-2):83-88. doi:10.1023/A:1012789819926/METRICS

82. Cassis LA, Marshall DE, Fettinger MJ, Rosenbluth B, Lodder RA. Mechanisms contributing to angiotensin II regulation of body weight. *American Journal of Physiology-*

Endocrinology and Metabolism. 1998;274(5):E867-E876. doi:10.1152/ajpendo.1998.274.5.E867

83. Fabris B, Candido R, Bortoletto M, et al. Dose and time-dependent apoptotic effects by angiotensin II infusion on left ventricular cardiomyocytes. *J Hypertens*. 2007;25(7):1481-1490. doi:10.1097/HJH.0B013E328121AAE7

84. Zou LX, Imig JD, Von Thun AM, Hymel A, Ono H, Navar LG. Receptor-Mediated Intrarenal Angiotensin II Augmentation in Angiotensin II–Infused Rats. *Hypertension*. 1996;28(4):669-677. doi:10.1161/01.HYP.28.4.669

85. Anke J, Van Eekelen M, Phillips MI. Plasma angiotensin II levels at moment of drinking during angiotensin II intravenous infusion. *American Journal of Physiology-Regulatory, Integrative and Comparative Physiology*. 1988;255(3):R500-R506. doi:10.1152/ajpregu.1988.255.3.R500

86. Wu R, Laplante MA, De Champlain J. Prevention of angiotensin II-induced hypertension, cardiovascular hypertrophy and oxidative stress by acetylsalicylic acid in rats. *J Hypertens*. 2004;22(4):793-801. doi:10.1097/01.hjh.0000098277.36684.6b

87. Herizi A, Jover B, Bouriquet N, Mimran A. Prevention of the Cardiovascular and Renal Effects of Angiotensin II by Endothelin Blockade. *Hypertension*. 1998;31(1):10-14. doi:10.1161/01.HYP.31.1.10

88. Leenen FHH, White R, Yuan B. Isoproterenol-induced cardiac hypertrophy: role of circulatory versus cardiac renin-angiotensin system. *American Journal of Physiology-Heart and Circulatory Physiology*. 2001;281(6):H2410-H2416. doi:10.1152/ajpheart.2001.281.6.H2410

89. Ennis IL, Escudero EM, Console GM, et al. Regression of Isoproterenol-Induced Cardiac Hypertrophy by Na+/H+ Exchanger Inhibition. *Hypertension*. 2003;41(6):1324-1329. doi:10.1161/01.HYP.0000071180.12012.6E

90. Kitagawa Y, Yamashita D, Ito H, Takaki M. Reversible effects of isoproterenol-induced hypertrophy on in situ left ventricular function in rat hearts. *American Journal of Physiology-Heart and Circulatory Physiology*. 2004;287(1):H277-H285. doi:10.1152/ajpheart.00073.2004

91. Murad N, Tucci PJ. Isoproterenol-Induced Hypertrophy May Result In Distinct Left Ventricular Changes. *Clin Exp Pharmacol Physiol*. 2000;27(5-6):352-357. doi:10.1046/j.1440-1681.2000.03254.x

92. Nagano M, Higaki J, Nakamura F, et al. Role of cardiac angiotensin II in isoproterenol-induced left ventricular hypertrophy. *Hypertension*. 1992;19(6):708-712. doi:10.1161/01.HYP.19.6.708

93. Brand T, Sharma HS, Schaper W. Expression of nuclear proto-oncogenes in isoproterenol-induced cardiac hypertrophy. *J Mol Cell Cardiol*. 1993;25(11):1325-1337. doi:10.1006/jmcc.1993.1145

94. Al-Rasheed NM, Al-Oteibi MM, Al-Manee RZ, et al. Simvastatin prevents isoproterenol-induced cardiac hypertrophy through modulation of the JAK/STAT pathway. *Drug Des Devel Ther*. 2015;9:3217-3229. doi:10.2147/DDDT.S86431

95. Chowdhury D, Tangutur AD, Khatua TN, Saxena P, Banerjee SK, Bhadra MP. A proteomic view of isoproterenol induced cardiac hypertrophy: Prohibitin identified as a potential biomarker in rats. *J Transl Med*. 2013;11(1):130. doi:10.1186/1479-5876-11-130

96. Takeshita D, Shimizu J, Kitagawa Y, et al. Isoproterenol-Induced Hypertrophied Rat Hearts: Does Short-Term Treatment Correspond to Long-Term Treatment? *The Journal of Physiological Sciences*. 2008;58(3):179-188. doi:10.2170/PHYSIOLSCI.RP004508

97. Boluyt MO, Long X, Eschenhagen T, et al. Isoproterenol infusion induces alterations in expression of hypertrophy-associated genes in rat heart. *American Journal of Physiology-Heart and Circulatory Physiology*. 1995;269(2):H638-H647. doi:10.1152/ajpheart.1995.269.2.H638

98. Golomb E, Abassi ZA, Cuda G, et al. Angiotensin II maintains, but does not mediate, isoproterenol-induced cardiac hypertrophy in rats. *American Journal of Physiology-Heart and Circulatory Physiology*. 1994;267(4):H1496-H1506. doi:10.1152/ajpheart.1994.267.4.H1496

99. Miyoshi T, Nakamura K, Miura D, et al. Effect of LCZ696, a dual angiotensin receptor neprilysin inhibitor, on isoproterenol-induced cardiac hypertrophy, fibrosis, and hemodynamic change in rats. *Cardiol J*. 2019;26(5):575-583. doi:10.5603/CJ.A2018.0048

100. Hanada K, Asari K, Saito M, Kawana J ichi, Mita M, Ogata H. Comparison of pharmacodynamics between carvedilol and metoprolol in rats with isoproterenol-induced cardiac hypertrophy: Effects of carvedilol enantiomers. *Eur J Pharmacol*. 2008;589(1-3):194-200. doi:10.1016/J.EJPHAR.2008.04.055

101. Grimm D, Holmer SR, Riegger GAJ, Kromer EP. Effects of Beta-Receptor Blockade and Angiotensin II Type I Receptor Antagonism in Isoproterenol - Induced Heart Failure in the Rat. *Cardiovascular Pathology*. 1999;8(6):315-323. doi:10.1016/S1054-8807(99)00021-6

102. Goyal BR, Mehta AA. Benefi cial role of spironolactone, telmisartan and their combination on isoproterenol-induced cardiac hypertrophy. *Acta Cardiol*. 2012;67(2):203-211. doi:10.1080/AC.67.2.2154211

103. Kaddoura S, Firth JD, Boheler KR, Sugden PH, Poole-Wilson PA. Endothelin-1 Is Involved in Norepinephrine-Induced Ventricular Hypertrophy in Vivo Acute Effects of Bosentan, an Orally Active, Mixed Endothelin ETA and ETB Receptor Antagonist. *Circulation*. 1996;93(11):2068-2079. doi:10.1161/01.CIR.93.11.2068/FORMAT/EPUB

104. Ficai S, Herizi A, Mimran A, Jover B. Endothelin Blockade In Angiotensin Ii Hypertension: Prevention And Treatment Studies In The Rat. *Clin Exp Pharmacol Physiol*. 2001;28(12):1100-1103. doi:10.1046/J.1440-1681.2001.03568.X

105. Park JB, Schiffrin EL. Cardiac and vascular fibrosis and hypertrophy in aldosterone-infused rats: role of endothelin-1. *Am J Hypertens*. 2002;15(2):164-169. doi:10.1016/S0895-7061(01)02291-9

106. KOBAYASHI M, MACHIDA N, TANAKA R, YAMANE Y. Effects of .BETA.-Blocker on Left Ventricular Remodeling in Rats with Volume Overload Cardiac Failure. *Journal of Veterinary Medical Science*. 2008;70(11):1231-1237. doi:10.1292/jvms.70.1231

107. Murray DB, McMillan R, Brower GL, Janicki JS. ET_A selective receptor antagonism prevents ventricular remodeling in volume-overloaded rats. *American Journal of Physiology-Heart and Circulatory Physiology*. 2009;297(1):H109-H116. doi:10.1152/ajpheart.00968.2008

108. Murray DB, Gardner JD, Brower GL, Janicki JS. Effects of nonselective endothelin-1 receptor antagonism on cardiac mast cell-mediated ventricular remodeling in rats. *American Journal of Physiology-Heart and Circulatory Physiology*. 2008;294(3):H1251-H1257. doi:10.1152/ajpheart.00622.2007

109. Lee DS, Kim DK, Choi SM, Kim YK, Ko BH, Jung YW. Bosentan Attenuates Compensatory Left Ventricular Hypertrophy Induced by Aortocaval Fistula in Rats. *Korean Circ J*. 2005;35(9):665. doi:10.4070/kcj.2005.35.9.665

110. Francis B, Winaver J, Karram T, Hoffman A, Abassi Z. Renal and Systemic Effects of Chronic Blockade of ETA or ETB Receptors in Normal Rats and Animals with Experimental Heart Failure. *J Cardiovasc Pharmacol*. 2004;44(Supplement 1):S54-S58. doi:10.1097/01.fjc.0000166214.42791.f2

111. Leskinen H, Vuolteenaho O, Ruskoaho H. Combined Inhibition of Endothelin and Angiotensin II Receptors Blocks Volume Load–Induced Cardiac Hormone Release. *Circ Res*. 1997;80(1):114-123. doi:10.1161/01.RES.80.1.114

112. Ross J, McCullagh WH. Nature of Enhanced Performance of the Dilated Left Ventricle in the Dog during Chronic Volume Overloading. *Circ Res*. 1972;30(5):549-556. doi:10.1161/01.RES.30.5.549

113. Badke F, Covell J, Covell JW, of Medicine P. Early changes in left ventricular regional dimensions and function during chronic volume overloading in the conscious dog. *Circ Res*. 1979;45(3):420-428. doi:10.1161/01.RES.45.3.420

114. Hayya J, Armstrong D, Gressis N. Management Science A Note on the Ratio of Two Normally Distributed Variables. Published online 1975. doi:10.1287/mnsc.21.11.1338
