# Supplementary figures and images for: Contributions of mechanical loading and hormonal changes to eccentric hypertrophy during volume overload: A Bayesian analysis using logic-based network models
Source: PLoS Comput Biol. 2025 Apr 16;21(4):e1012390. doi: 10.1371/journal.pcbi.1012390 (PMC12040246; doi:10.1371/journal.pcbi.1012390)

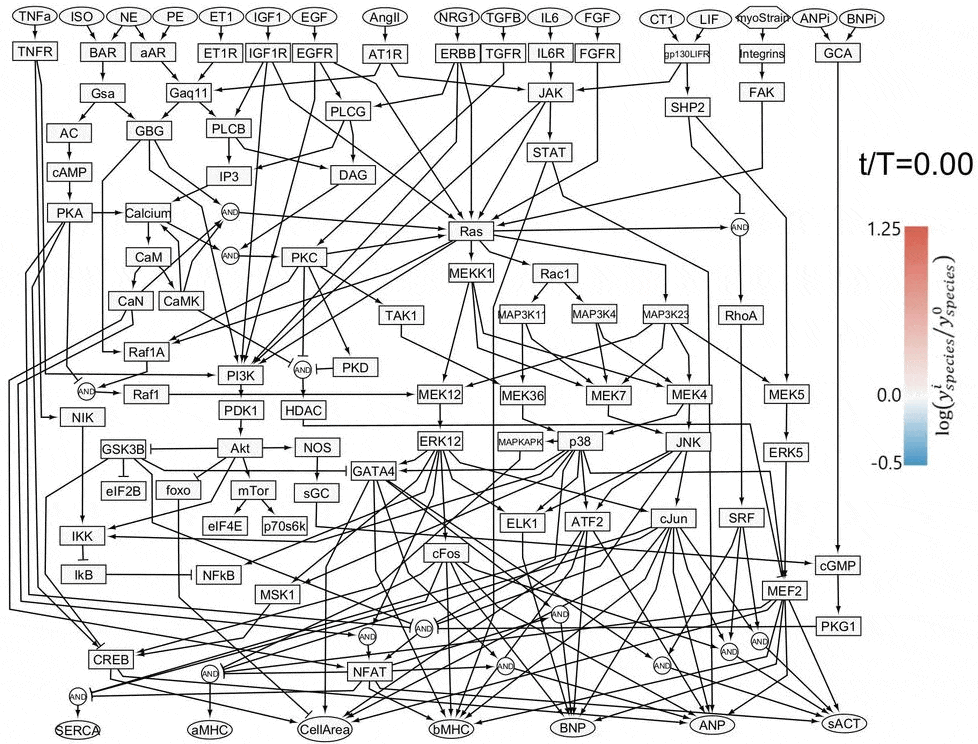

Supplement: S2 Material — Red colors indicate a higher level of activity during VO relative to baseline, while blue colors indicate a lower level of activity during VO relative to baseline. (GIF) [file pcbi.1012390.s003.gif]
