# Supplementary material for: Contributions of mechanical loading and hormonal changes to eccentric hypertrophy during volume overload: A Bayesian analysis using logic-based network models
Source: PLoS Comput Biol. 2025 Apr 16;21(4):e1012390. doi: 10.1371/journal.pcbi.1012390 (PMC12040246; doi:10.1371/journal.pcbi.1012390)
Supplement: Attachment — Submitted filename: BracamonteR1_ReviewerResponse_2412.docx [file pcbi.1012390.s005.docx]

Dear Dr. Holmes,

Thank you very much for submitting your manuscript "Contributions of mechanical loading and hormonal changes to eccentric hypertrophy during volume overload: a Bayesian analysis using logic-based network models." for consideration at PLOS Computational Biology.

As with all papers reviewed by the journal, your manuscript was reviewed by members of the editorial board and by several independent reviewers. In light of the reviews (below this email), we would like to invite the resubmission of a significantly-revised version that takes into account the reviewers' comments.

I am submitting the decision to request major revisions because I believe that the methodology developed here is novel and powerful and the application is highly innovative. That said, please note that both reviewers raised substantial concerns, with one reviewer recommending rejection. Addressing these reviewer concerns will greatly improve the potential impact of your work.

We cannot make any decision about publication until we have seen the revised manuscript and your response to the reviewers' comments. Your revised manuscript is also likely to be sent to reviewers for further evaluation.

When you are ready to resubmit, please upload the following:

[1] A letter containing a detailed list of your responses to the review comments and a description of the changes you have made in the manuscript. Please note while forming your response, if your article is accepted, you may have the opportunity to make the peer review history publicly available. The record will include editor decision letters (with reviews) and your responses to reviewer comments. If eligible, we will contact you to opt in or out.

[2] Two versions of the revised manuscript: one with either highlights or tracked changes denoting where the text has been changed; the other a clean version (uploaded as the manuscript file).

Important additional instructions are given below your reviewer comments.

Please prepare and submit your revised manuscript within 60 days. If you anticipate any delay, please let us know the expected resubmission date by replying to this email. Please note that revised manuscripts received after the 60-day due date may require evaluation and peer review similar to newly submitted manuscripts.

Thank you again for your submission. We hope that our editorial process has been constructive so far, and we welcome your feedback at any time. Please don't hesitate to contact us if you have any questions or comments.

Sincerely,

Daniel A Beard

Section Editor

PLOS Computational Biology

Figure Files:

While revising your submission, please upload your figure files to the Preflight Analysis and Conversion Engine (PACE) digital diagnostic tool, https://pacev2.apexcovantage.com. PACE helps ensure that figures meet PLOS requirements. To use PACE, you must first register as a user. Then, login and navigate to the UPLOAD tab, where you will find detailed instructions on how to use the tool. If you encounter any issues or have any questions when using PACE, please email us at figures@plos.org.

Data Requirements:

Please note that, as a condition of publication, PLOS' data policy requires that you make available all data used to draw the conclusions outlined in your manuscript. Data must be deposited in an appropriate repository, included within the body of the manuscript, or uploaded as supporting information. This includes all numerical values that were used to generate graphs, histograms etc.. For an example in PLOS Biology see here: http://www.plosbiology.org/article/info%3Adoi%2F10.1371%2Fjournal.pbio.1001908#s5.

Reproducibility:

To enhance the reproducibility of your results, we recommend that you deposit your laboratory protocols in protocols.io, where a protocol can be assigned its own identifier (DOI) such that it can be cited independently in the future. Additionally, PLOS ONE offers an option to publish peer-reviewed clinical study protocols. Read more information on sharing protocols at https://plos.org/protocols?utm_medium=editorial-email&utm_source=authorletters&utm_campaign=protocols

The authors would like to thank the Editor and Reviewers for their time, valuable feedback, and the opportunity to improve and resubmit the manuscript. As requested, we carefully addressed the Reviewers’ comments in the manuscript and supplementary material; those revisions included the addition of extensive new comparison data for validation, a new approach to presenting model results and comparison data in the figures, and complete rewriting of several sections of the manuscript and supplement. Point-by-point responses are provided below. Associated revisions are identified using red text or strike throughs in the “Changes Highlighted” version of the revised manuscript.

**Reviewer #1:**

The manuscript from Bracamonte et al. is very interesting and provides some notable findings:

First, it highlights the important role of Endothelin1 receptor activity in driving hypertrophic responses.

Second, it provides a plausible explanation for the suppression of mechano-transduction signaling in experimental volume overload (VO).

This challenges the common assumption that VO hypertrophy is driven by myocyte overstretch, as used in most computational models.

Additionally, though less supported by results and data, the authors claim that the model can replicate drug responses not used in its calibration and predicts that combining ERA with ARB may effectively reduce cardiomyocyte hypertrophy and dysfunction in VO.

Overall, the paper offers significant insights, especially for the computational modeling community.

However, several concerns must be addressed before it can be considered for publication in PLOS Computational Biology

We thank the Reviewer for the assessment of our work as interesting and providing notable findings and insights, especially for the computational modeling in biology community. We have sought to fully address the concerns identified below, with particular attention to improved validation of the model against experimental data.

As part of our response to the concerns raised by Reviewer 2, and to better focus on more extensively validating the calibrated model against available experimental data, we decided to eliminate sections 2.9 and 3.6, which contained the discussion on the predicted impact of ERA + ARB during volume overload.

1) While the abstract and introduction are clear and well-written, the presentation of the methods and results in the manuscript is somewhat sloppy, making it difficult to follow and understand. There are multiple instances where figure labels are incorrect, variable names in equations are inconsistent, and reference citations are either inconsistent or missing from the References list. The manuscript sometimes feels like an early draft and will require thorough proofreading and revision in this regard.

For example (a partial list of such issues):

*) Lines 148, 158: LVM/BM or LVM/BW

All references to normalization of left ventricular mass to body mass in text, captions and figures are now consistently expressed as LVM/BM.

*) Line 204: is i an index for a given time?

Yes, the superindex i is an index for a given time, while the superindex 0 is reserved for the baseline state prior to the induction of VO. This is now explicitly explained in the manuscript after its first use in Equation (4), line 279.

*) Line 214: what is myoStrain?

The network model provides an input labeled myoStrain as a generic representation of the stretch-activated signaling pathways thought to be involved in transducing changes in myocyte mechanics into hypertrophy, and is now defined in lines 199-207 in the revised manuscript. As with all inputs of the cardiomyocyte network model, it is a normalized variable ranging from 0 to 1, which is calculated from left ventricular mechanical strain using a mapping function. The new section 2.4.3. (lines 269-288) in the revised manuscript provides more detailed information on myoStrain and the mapping function.

*) CellArea is introduced in l 288, Is "Cell Area" above on page 18, l 271,276 the same?

Yes, in the original manuscript “CellArea” and “Cell Area” refer to the same variable, one of the output nodes of the cardiomyocyte network. In the revised manuscript, this variable is consistently labeled “CellArea” in text, equations and figures.

*) l346 "and" missing

We eliminated this whole section in the new version of the manuscript. We performed a detailed grammar check in order to eliminate other similar errors.

*) l388: should be 0.05 < w < 0.06, at least according to S1.5

Thanks for pointing this out. It was corrected to 0.05 < w < 0.06, and now appears in line 526.

*) Figure 4 and 5 seem to be switched

We corrected our figure numbering throughout the text.

*) l 413: I don't really see that from Figure S1.11. Both network representations have the label S1.11 in the supplement.

Figure S1.11 is not included in this new version, as a result of a reduced emphasis on pharmacological treatment of VO in response to feedback from Reviewer 2.

*) l 428: "... of ANP, BNP, and bMHC (Fig 5)." -> Figure 6?

The whole validation section, including figure 5, were completely reformulated. In this new version we made sure figures were called in the correct order.

*) l.476 Delley et al. missing reference number in [] (This is the case for many references in the manuscript). Griffin et al. is missing in the reference list.

*) l.637, reference numbers missing; I don't see a Dell'Italia 1995 paper

We thank we reviewer for pointing out this issue with our reference list. We revised all references to make sure they are appropriately cited and listed in the main text and supplementary material S1.

*) From S6 to S7: are the superscripts removed here on purpose?

Supplementary section *S1.3. Estimation of time-varying probability distributions of stretch* was completely reformulated; equations S1.1 to S1.4 now include superscripts where required.

*) S8, S9: not all the variables used in these Equations are introduced.

Equations S8 and S9 are now Equations S1.2 and S1.3; all variables in the equations are defined in the paragraph following the equations.

*) "to estimate the likelihood of the solution (Figure S1.1b)." should be S1.2b

We corrected our figure numbering throughout the text and supplementary material.

*) S10: "The mechanical strain is to the network input myoStrain with an exponential function:"

A word ("mapped"?) is missing.

Yes, the word mapped was missing. As part of the revision, we rewrote this entire section, which also corrected this error.

2) The strain computation outlined in the manuscript is not very clear.

In particular Lines 214-215 and Eq (2) have to be improved. Where does this come from, what are the different variables?

The supplemental material helps a bit but chapter S1.3 should be revised as well.

Figure S1.3: " [...] estimation of stretch/stress probability" stretch/strain?

Both reviewers asked about the strain calculations. In response, we completely rewrote Section 2.4, and the sections that discuss the strain calculation are reproduced below for your review. In addition, we completely rewrote Section S1.3 in the supplemental materials and devised a new set of associated figures to illustrate the steps involved in producing a time-varying probability distribution of expected myocardial stretch values over the course of experimental volume overload. Due to its length, we did not reproduce Section S1.3 here.

2.4.1. Calculating end-diastolic strain relative to an unloaded state.

Let the circumference of a mechanically unloaded sphere (i.e., floating in saline without any applied pressure) be $2\pi r_{0}$, where r is the radius of the sphere and the subscript zero indicates the unloaded or reference state. Once inflated to end-diastole, the sphere’s circumference is $2\pi r_{ED}$. The stretch $(\lambda)$ around its circumference is defined as the ratio of loaded to unloaded length:

$$\lambda=\frac{2\pi r_{ED}}{2\pi r_{0}}=\frac{r_{ED}}{r_{0}}$$

(1)

In a spherical model of the LV, myocytes with any orientation parallel to the surface will also stretch by this same fraction. While an actual heart is never fully unloaded unless it is arrested and removed from the body, most mechanics models of growth use the unloaded state as a mathematical reference point because it is easy to define and doesn’t change with beat-to-beat variations in hemodynamics.

Since many studies reviewed here tracked changes in volumes, we found it convenient to express stretch and strain in terms of volumes rather than radius. Recognizing that the volume of a sphere is proportional to $r^{3}$, we can rewrite the equation for stretch as:

$$\lambda=\frac{r_{ED}}{r_{0}}=\left( \frac{V_{ED}}{V_{0}} \right)^{1/3}$$

(2)

Finally, for large deformations measured relative to a fixed reference state, the most appropriate measure of strain is the Lagrangian strain [66]:

$$\varepsilon_{f}=\frac{1}{2}\left( \lambda^{2}-1 \right)=\frac{1}{2}\left( \left( \frac{V_{ED}}{V_{0}} \right)^{2/3}-1 \right)$$

(3)

2.4.2. Effect of growth on end-diastolic strain.

Returning to the unloaded sphere, if each myocyte grows longer by adding sarcomeres in series – as occurs during eccentric hypertrophy – then the unloaded radius $r_{0}$ and volume $V_{0}$ will be larger, and the stretch at a given end-diastolic volume will be smaller (Equation 3). In other words, growth changes the amount of stretch each myocyte experiences, which alters the stimulus for further growth. One of the key features of the model presented here is that it accounts for this critical feedback between growth and the hypertrophic signaling that produces it. Figure 2a illustrates this point graphically. In a spherical model of a normal heart, stretch increases as a nonlinear function of volume during simulated inflation. Imposing a sudden volume overload produces a big increase in end-diastolic volume (blue circle) relative to baseline (black circle). A modest amount of growth (10% volume increase) shifts the entire curve rightward, so that stretch is lower at a given volume, and inflation to a larger volume is required to achieve the same stretch (red triangle).

A thin-walled sphere is a very simplistic representation of the geometry of the left ventricle, and a more sophisticated geometric model would produce a different quantitative relationship between volume and stretch. But that was not an important limitation for this study, for three reasons. First, the spherical model does remarkably well when compared to actual sarcomere lengths measured by Ross et al. in normal and hypertrophied hearts fixed at controlled volumes (Figure 2b), suggesting its use here is reasonable. Second, the trends we predict here – an acute increase in end-diastolic strain followed by a gradual decline to baseline levels at 6 weeks – were experimentally verified by Emery and Omens (1997) using markers attached to the LV surface [68]. Finally, it is important to note that the stretch and strain values we calculate from the spherical model (or any alternative model) are ultimately mapped onto a normalized input to the signaling network, and the relative influence of that input is calibrated against data (see below). Therefore, a different geometric model would just produce a different calibration; the overall behavior of the system would not be affected.

Consistent with the approach described above for integrating data on neurohormonal alterations, we estimated the time-varying PDF of strain by integrating the spherical model with data from sixteen articles reporting changes in LVM [15,20–34] and twenty-one articles reporting changes in $V_{ED}$ during experimental MR in dogs, as well as five articles reporting end-diastolic myocardial or sarcomere stretch relative to baseline in healthy dogs [67,69–72].

2.4.3. Mapping end-diastolic strain onto the network input myoStrain.

The mechanical input to the cardiomyocyte network model (myoStrain) is a normalized variable ranging from 0 to 1 (Section 2.5). However, in practice, increasing this input from its default baseline of 0.06 to just 0.2 produces the maximum possible CellArea output (CellArea=1) in the published version of the model employed here [73]. Thus, we sought to design a function that maps the range of expected values for organ-scale end-diastolic strain at baseline or during volume overload into myoStrain input values within the 0-0.2 working range. We also wanted the mapping function to produce a zero myoStrain input when organ-scale strain was zero (i.e., when the LV is in a completely unloaded state). We decided to use an exponential mapping function of the form:

$$w_{myoStrain}^{i}=C_{myo}\left( e^{D_{myo} \varepsilon_{f}^{i}/\varepsilon_{f}^{0}}-1 \right)$$

(4)

where superindex i refers to any given time, while superindex 0 is reserved for the basline (pre-VO) state, $\varepsilon_{f}$ and $w_{myoStrain}$ are the maximum fiber strain and its corresponding mapped myoStrain, and $C_{myo}$and $D_{myo}$ are mapping parameters to be fitted by a Markov Chain Monte Carlo (MCMC) algorithm (Section 2.6). To reduce the order of the MCMC parameter space, we fixed the baseline myoStrain weight $\left( w_{myoStrain}^{0} \right)$ at a single value for each MCMC run and repeated the process for $w_{myoStrain}^{0}$ values of 0.02, 0.04, 0.05 0.055, 0.06, 0.065, 0.07, 0.08, and 0.09. On each MCMC iteration we assign a random value to $C_{myo}$ and calculate $D_{myo}$ with equation (4), the prescribed value of $w_{myoStrain}^{0}$, and reported baseline values of strain $\left( \varepsilon_{f}^{0} \right)$ [67,69–72].

3) "The PDF display the expected trends over time, that is, an acute increase in strain owing to the sudden increase of Ved, followed by a gradual decrease driven by the compensatory hypertrophic response (Fig 3)." I somehow struggle to see that behavior in Figure 3. I see an increase in Ved in 3b, maybe not a "sudden increase" and strain starts at approx. 1.2 in 3c) Is this the acute increase? If so, the figure should be improved to make this clearer.

We agree that the acute changes were difficult to see in the original figures. We added an inset window to figures 3a,3b, and 3c showing how the variables LVM, V_ED_ and strain change at very early times, in the normalized time-range $0<t/\tau<0.1$. We believe the insets better show the sharp acute increase in V_ED_ and strain, vs. the gradual rise in LV mass.

4) The validation is not very convincing, in particular Fig 7c, CellArea fold change. Most data points are outside the 67% confidence interval and validating a model built on rats and dogs with data from mice is not optimal. Maybe there are more experimental data to strengthen this part of the study.

We agree that the validation section of the original manuscript needed improvement. In the revised manuscript, we have clarified exactly what aspects of the model are being validated and how we assess whether the model-predicted range of responses does or does not agree with the available data. As suggested, we also compiled substantially more experimental data from rats and dogs for comparison and excluded data from mice. Overall, the revised manuscript validates model predictions against a total of 56 experimental datasets (mean±SD) from 43 independent studies of infusion of agonists that induce myocyte hypertrophy, alone or in combination with receptor blockers used clinically to treat heart failure in rats or dogs. Our revised approach to validation is described in detail in a completely rewritten methods section on validation (Section 2.8) and is also reflected in a completely new format for presenting and comparing model predictions to data in Figures 5 and 6. In those new figures, we first illustrate how the Bayesian approach produces a range of different predictions for any output, and how we translate the distribution of predicted outcomes into boxplots (Figure 5). We then assess whether >75% of predictions agree with qualitative reports (increased / no change / decreased) from experiments (Figure 5) and also how many experimental means fall within model-predicted 50% and 90% confidence intervals (Figure 6). Overall, 50 of 56 (89%) experimentally observed mean changes in LVM/BM across a range of drug administration protocols fell within the 90% confidence intervals predicted by the model. We consider this a striking result given that the model was never explicitly calibrated for any of those interventions.

5) Furthermore, the model's ability to replicate drug responses and thus its predictive quality are only partially supported by the data and results. The changes shown in Figure 8a differ significantly in magnitude from the experimental data. In Figure 8b, it is unclear whether the changes in VO+ARB are statistically significant given the error bars. Additionally, in Figure 8c, the model fails to replicate the significant increase observed at t/tau = 2.7.

The original Figure 8 (now Figure 7 in the revised manuscript) focused on simulations of drug treatment during experimental volume overload. We agree with the reviewer that the network model presented here omits secondary hemodynamic effects of many commonly used cardiovascular drugs that are central to their action. In fact, our next planned step is to integrate the calibrated network model presented here with a mechanical model of the heart and circulation to yield a multiscale model capable of capturing a greater range of interactions between hormones, drugs, and mechanics during VO. In the revised manuscript, we therefore treat the simulations of volume overload plus drugs as exploratory rather than as validations and have completely rewritten the validation sections (Methods Section 2.8, Results Section 3.4, and Discussion) accordingly.

In the revised manuscript, we introduced new Methods (Section 2.9) and Results (Section 3.5) sections titled “Administration of receptor blockers during VO” that describe these exploratory simulations. We explicitly state throughout the text that these simulations are exploratory and intended to evaluate the capabilities and limitations of the current version of the model (lines 468-470, 663-664, 742-745, 842-854).

Despite the limitations of the signaling network, in the new Figure 7 it is apparent that the 90% confidence interval of the network model predictions encompasses all of the experimental means, and the 50% confidence interval encompasses many of them. The biggest failure of the network model was in not capturing the reported increase in LV mass following long periods of beta blockade (Figure 7a), an effect we suspect is secondary to the suppression of left ventricular contractility by drug treatment. This is a good example of an effect that could only be captured in a multiscale model that incorporates drug effects on mechanics as well as signaling. For ARB, slightly more than half the simulations predicted a reduction in VO-induced hypertrophy compared to untreated VO (Figure 7b), which is consistent with the fact that 3 out of 5 studies reported a significant reduction while 2 found no effect. For ERA, the vast majority of model simulations predicted a reduction in hypertrophy, consistent with the fact that all 4 experimental studies reported a significant (though sometimes small) effect.

Minor:

*) for the sake of completeness (and its importance for the study) ET-1 should also be introduced around line 98

We thank the reviewer for the suggestion. We now introduce ET1 in the introduction (line 106), as part of the list of key circulating neurohormonal factors considered in this study.

**Reviewer #2:**

The authors combine data from 70 studies "on experimental volume overload (VO) in dogs and rats. This paper extracts data from these various papers to create a statistical models using data extracted from various papers. The authors claim the model "reproduces a number of responses to drug therapy not used in its calibration, and predicts that a combination of endothelin receptor antagonist and angiotensin receptor blockers would have the greatest potential to dampen cardiomyocyte hypertrophy and dysfunction in VO".

Assessment

Strengths

Mitral regurgitation continues to be a significant clinical problem, despite the recent advances in valve repair and replacement. So, there might be some interest in this study.

Weaknesses

With effective valve replacement procedures in place, interest in treating cardiac disease associated with mitral regurgitation with various medications is of limited interest.

We agree with the reviewer that mitral valve repair or replacement is the definitive treatment for mitral regurgitation (MR), the only one capable of not only arresting the progression of ventricular dilation and development of heart failure, but in some cases partially reversing this process. We never intended to suggest this condition should be managed solely with medication, and have substantially revised the manuscript in order to de-emphasize the discussion regarding novel drug combinations and focus squarely on what we believe to be the key contributions here: the potential to predict the integrated effects of hormones/drug (chemical) and devices (mechanical) and the probabilistic nature of those predictions. Those revisions included completely deleting the sections original titled “Screening of pharmacological alternatives in the setting of VO” from both Methods (original section 2.9) and Results (original section 3.5), as well as any discussion or conclusions involving those results.

We also substantially revised the Discussion to clarify our motivation for this study (lines 729-738): “Our primary motivation in developing a systems-level model of hypertrophy during VO is that clinical conditions such as mitral regurgitation trigger changes in multiple hypertrophic stimuli, including myocyte stretch/stress as well as circulating levels of hormones known to modulate hypertrophy. Furthermore, most patients receive multiple therapies – such as administration of β-blockers and mitral valve repair or replacement – that themselves modify different hypertrophic stimuli. Thus, predicting the effects of any potential treatment or combination of treatments requires a model that can anticipate the crosstalk amongst relevant signaling pathways. In this respect, the validations shown here against experiments where agonist infusion was combined with a receptor blocker (Fig 6d) are particularly promising.”

In this study, we validated the model against the experiments that were available in the literature, most of which involved drugs. However, in the future we intend to use this model not to develop novel drug therapies but rather to tackle a critical question that arises in the management of patients every day. In patients with MR as well as with other conditions such as congenital heart disease, *the key question is not how but when to intervene*. Surgery carries risk, and surgeons do not want to subject a patient to those risks unless/until it is absolutely necessary. Yet for many of these conditions, waiting also poses a risk: that cardiac function will deteriorate to the point that normal function can no longer be fully restored by treatment. Balancing these two considerations is very difficult, and even metrics and indices that show predictive power on average fail many individuals. For the specific case of mitral valve repair, guidelines suggest operating before ejection fraction (EF) falls below 60%, but in our studies (authors LJD, BP), 20% of patients treated according to guidelines nevertheless suffered impaired cardiac function post-operatively, and in retrospect would have likely done better with earlier intervention. We believe that realistically complex, multifactorial models of hypertrophy are a prerequisite to developing patient-specific models for clinical decision support.

There are many studies included. To make any sense of data from different studies it is, in my opinion, imperative that the conditions between the studies be comparable (time course, disease severity, age of animals, gender etc etc) and carefully considered. There is insufficient information and discussion provided by the authors demonstrating how and whether the studies were comparable.

There are many ways to introduce volume overload and the severity depends on many factors. There is no discussion in the paper on how issues related to model diversity and complexity were addressed. Without careful consideration for technical differences between the various studies, it is difficult to understand how anything meaningful can come from simply pooling data from these many studies.

Large-scale systems biology models like the one presented here aim to address a fundamental challenge facing biomedical research today and highlighted by your question: after compiling so much data over many years, how do we make sense of it while counteracting the now-recognized problems with reproducibility and other limitations of individual, relatively small studies? The reason we are so excited about the approach presented here is that it provides a way to address this challenge by generating something analogous to a meta-analysis of available experimental data on a condition and its treatment, with the additional benefit that a large body of knowledge about the known biology of the system is incorporated as prior knowledge in the analysis, through the structure of the computational model.

The animal studies reviewed here all started with physiological normal animals, introduced levels of volume overload that would be considered moderately severe or severe by clinical standards, and stopped (for ethical reasons) before reaching end-stage heart failure. But they are diverse in other ways, combining different species and in some cases different sexes (which is not always reported). We completely agree that it is not obvious whether it is possible to combine these data into a meaningful predictive model, and if so then we must provide convincing proof. In this respect the first version of the manuscript clearly failed, because neither reviewer was convinced.

In the revised manuscript, we revamped our approach to model validation, compiling substantially more experimental data for comparison, better defining metrics for assessing whether predictions agree with experiments, rewriting all text related to validation, and completely reworking all relevant figures. The revised manuscript now calibrates the network model using data from 70 studies of volume overload and validates its predictions against a total of 56 experimental datasets (mean±SD) from 43 independent studies of infusion of hypertrophic agonists alone or in combination with various heart failure drugs. Our revised approach to validation is now described in completely rewritten Methods (Section 2.8) and Results (Section 3.4) sections and in new Figures 5 and 6. In those new figures, we first illustrate how the Bayesian approach produces a range of different predictions for any output, and how we translate the distribution of predicted outcomes into boxplots (Figure 5). We then assess whether >75% of predictions agree with qualitative reports (increased / no change / decreased) from experiments (Figure 5) and also how many experimental means fall within model-predicted 50% and 90% confidence intervals (Figure 6). Overall, 50 of 56 (89%) experimentally observed mean changes in LVM/BM across a range of drug administration protocols fell within the 90% confidence intervals predicted by the model. We consider this a striking result given that the model was never explicitly calibrated for any of those interventions.

In order to explicitly call attention to some of the limitations you point out here, we added the following text to the Limitations section (lines 835-841): “Another source of variability that would be more difficult to address is the fact that the data used to calibrate the model were gathered from studies in different animal models, performed by different groups. These studies typically began with healthy animals and introduced a severe or moderately severe overload at a known point in time. However, factors such as variations in overload severity, species differences, and sex differences likely contributed to the variability in predictions in the final, calibrated model.”

The method for estimating the myocardial strain is unlikely to be valid. In the end, strain must be measured dynamically and using interventions that monitor chamber or muscle deformation as a function of an applied changes in force or pressure. I have no confidence that the method used is in this paper is meaningful.

Both reviewers asked about the strain calculations. In response, we completely rewrote Section 2.4, and the sections that discuss the strain calculation are reproduced below for your review. In addition, we completely rewrote Section S1.3 in the supplemental materials and devised a new set of associated figures to illustrate the steps involved in producing a time-varying probability distribution of expected myocardial stretch values over the course of experimental volume overload. Due to its length, we did not reproduce Section S1.3 here.

Regarding confidence, it is hard to know how to address this comment given that no specific concerns were raised. Beyond the text provided below and in the revised manuscript, hopefully it will help to know that the senior author of this study (JWH) has spent his entire career measuring strains in the heart and other tissues, using nearly every known method: implanted beads filmed with x-rays, painted or embedded markers imaged with high-speed video, speckle-tracking, sonomicrometry, 2D and 3D ultrasound, and CINE-DENSE MRI. He has also taught undergraduate and graduate courses and international workshops on soft tissue mechanics and measurement of stresses and strains in the heart and other soft tissues. By comparison, the equations used here are quite simple and can be proven to be mathematically correct.

2.4.1. Calculating end-diastolic strain relative to an unloaded state.

Let the circumference of a mechanically unloaded sphere (i.e., floating in saline without any applied pressure) be $2\pi r_{0}$, where r is the radius of the sphere and the subscript zero indicates the unloaded or reference state. Once inflated to end-diastole, the sphere’s circumference is $2\pi r_{ED}$. The stretch $(\lambda)$ around its circumference is defined as the ratio of loaded to unloaded length:

| $\lambda=\frac{2\pi r_{ED}}{2\pi r_{0}}=\frac{r_{ED}}{r_{0}}$ | Equation (1) |
| --- | --- |

In a spherical model of the LV, myocytes with any orientation parallel to the surface will also stretch by this same fraction. While an actual heart is never fully unloaded unless it is arrested and removed from the body, most mechanics models of growth use the unloaded state as a mathematical reference point because it is easy to define and doesn’t change with beat-to-beat variations in hemodynamics.

Since many studies reviewed here tracked changes in volumes, we found it convenient to express stretch and strain in terms of volumes rather than radius. Recognizing that the volume of a sphere is proportional to $r^{3}$, we can rewrite the equation for stretch as:

| $\lambda=\frac{r_{ED}}{r_{0}}=\left( \frac{V_{ED}}{V_{0}} \right)^{1/3}$ | Equation (2) |
| --- | --- |

Finally, for large deformations measured relative to a fixed reference state, the most appropriate measure of strain is the Lagrangian strain [66]:

| $\varepsilon_{f}=\frac{1}{2}\left( \lambda^{2}-1 \right)=\frac{1}{2}\left( \left( \frac{V_{ED}}{V_{0}} \right)^{2/3}-1 \right)$ | Equation (3) |
| --- | --- |

2.4.2. Effect of growth on end-diastolic strain.

Returning to the unloaded sphere, if each myocyte grows longer by adding sarcomeres in series – as occurs during eccentric hypertrophy – then the unloaded radius $r_{0}$ and volume $V_{0}$ will be larger, and the stretch at a given end-diastolic volume will be smaller (Equation 3). In other words, growth changes the amount of stretch each myocyte experiences, which alters the stimulus for further growth. One of the key features of the model presented here is that it accounts for this critical feedback between growth and the hypertrophic signaling that produces it. Figure 2a illustrates this point graphically. In a spherical model of a normal heart, stretch increases as a nonlinear function of volume during simulated inflation. Imposing a sudden volume overload produces a big increase in end-diastolic volume (blue circle) relative to baseline (black circle). A modest amount of growth (10% volume increase) shifts the entire curve rightward, so that stretch is lower at a given volume, and inflation to a larger volume is required to achieve the same stretch (red triangle).

A thin-walled sphere is a very simplistic representation of the geometry of the left ventricle, and a more sophisticated geometric model would produce a different quantitative relationship between volume and stretch. But that was not an important limitation for this study, for three reasons. First, the spherical model does remarkably well when compared to actual sarcomere lengths measured by Ross et al. in normal and hypertrophied hearts fixed at controlled volumes (Figure 2b), suggesting its use here is reasonable. Second, the trends we predict here – an acute increase in end-diastolic strain followed by a gradual decline to baseline levels at 6 weeks – were experimentally verified by Emery and Omens (1997) using markers attached to the LV surface [68]. Finally, it is important to note that the stretch and strain values we calculate from the spherical model (or any alternative model) are ultimately mapped onto a normalized input to the signaling network, and the relative influence of that input is calibrated against data (see below). Therefore, a different geometric model would just produce a different calibration; the overall behavior of the system would not be affected.

Consistent with the approach described above for integrating data on neurohormonal alterations, we estimated the time-varying PDF of strain by integrating the spherical model with data from sixteen articles reporting changes in LVM [15,20–34] and twenty-one articles reporting changes in $V_{ED}$ during experimental MR in dogs, as well as five articles reporting end-diastolic myocardial or sarcomere stretch relative to baseline in healthy dogs [67,69–72].

2.4.3. Mapping end-diastolic strain onto the network input myoStrain.

The mechanical input to the cardiomyocyte network model (myoStrain) is a normalized variable ranging from 0 to 1 (Section 2.5). However, in practice, increasing this input from its default baseline of 0.06 to just 0.2 produces the maximum possible CellArea output (CellArea=1) in the published version of the model employed here [73]. Thus, we sought to design a function that maps the range of expected values for organ-scale end-diastolic strain at baseline or during volume overload into myoStrain input values within the 0-0.2 working range. We also wanted the mapping function to produce a zero myoStrain input when organ-scale strain was zero (i.e., when the LV is in a completely unloaded state). We decided to use an exponential mapping function of the form:

| $w_{myoStrain}^{i}=C_{myo}\left( e^{D_{myo} \varepsilon_{f}^{i}/\varepsilon_{f}^{0}}-1 \right)$ | Equation (4) |
| --- | --- |

where superindex i refers to any given time, while superindex 0 is reserved for the basline (pre-VO) state, $\varepsilon_{f}$ and $w_{myoStrain}$ are the maximum fiber strain and its corresponding mapped myoStrain, and $C_{myo}$and $D_{myo}$ are mapping parameters to be fitted by a Markov Chain Monte Carlo (MCMC) algorithm (Section 2.6). To reduce the order of the MCMC parameter space, we fixed the baseline myoStrain weight $\left( w_{myoStrain}^{0} \right)$ at a single value for each MCMC run and repeated the process for $w_{myoStrain}^{0}$ values of 0.02, 0.04, 0.05 0.055, 0.06, 0.065, 0.07, 0.08, and 0.09. On each MCMC iteration we assign a random value to $C_{myo}$ and calculate $D_{myo}$ with equation (4), the prescribed value of $w_{myoStrain}^{0}$, and reported baseline values of strain $\left( \varepsilon_{f}^{0} \right)$ [67,69–72].

With respects to the biochemical measurements, it is not clear how many studies made measurements for the various factors incorporated into the model (such as adrenaline, Ang II, ANP etc etc). There was also no discussion or consideration for how and when these measurements were made in relationship to the time course of the volume overload condition (i.e. stage of the disease).

In the previous version, we provided tables in supplementary material S1.1. listing all data sources and indicating which animal model was used and which variables were collected. In the revised manuscript, we added a new table S1.2 containing similar information for all of the independent studies used to validate model predictions.

To specifically address the question of how many measurements were collected for specific model inputs or outputs, the revised manuscript specifies the number of experimental datapoints and the number of studies they were collected from, for all calibration and validation processes, in the following locations:

- The caption for Figure 1 specifies the number of reports (papers) and datasets (mean±SD for a given time or concentration) we used to determine the time-varying probability distributions of circulating neurohormone levels. The graphs in Figure 1 show how those data were distributed across time, and the symbols indicate which data were drawn from studies in rats vs. dogs.
- Section 2.4.2 specifies the number of articles reviewed to collect data for each variable involved in the estimation of myocardial stretch (lines 262-268).
- Section 2.6.1. specifies the number of studies (articles) and datasets collected and used to calibrate the network model (lines 356-362).
- In section 2.8.1. (lines 421-42316, 427-430, 432-433), we list the number of articles and datasets used to validate model predictions of responses to agonist infusion.
- In section 2.8.2. (lines 447-453) we list the number of articles and datasets used to validate model predictions of responses to agonist infusion plus receptor blocker administration.
- In section 3.5. (lines 470-478 ), we provide the number of studies and experimental datasets of receptor blocker administration in VO that were collected and used for exploration of the limitations of the model.

Have the authors made all data and (if applicable) computational code underlying the findings in their manuscript fully available?

The PLOS Data policy requires authors to make all data and code underlying the findings described in their manuscript fully available without restriction, with rare exception (please refer to the Data Availability Statement in the manuscript PDF file). The data and code should be provided as part of the manuscript or its supporting information, or deposited to a public repository. For example, in addition to summary statistics, the data points behind means, medians and variance measures should be available. If there are restrictions on publicly sharing data or code —e.g. participant privacy or use of data from a third party—those must be specified.

Reviewer #1: Yes

**Reviewer #2: No: I did not see a reference to this.**

Links to the GitHub repositories containing the MATLAB codes utilized to generate our results, and the tabulated experimental data used for calibration and validation were provided in lines 233 and 257 of the original manuscript. In this new version, those links are provided in lines 307 and 331.

PLOS authors have the option to publish the peer review history of their article (what does this mean?). If published, this will include your full peer review and any attached files.

If you choose “no”, your identity will remain anonymous but your review may still be made public.

Do you want your identity to be public for this peer review? For information about this choice, including consent withdrawal, please see our Privacy Policy.

Reviewer #1: No

Reviewer #2: No
